# USP22 promotes the proliferation and Sorafenib resistance of hepatocellular carcinoma cells via its deubiquitinase activity

**DOI:** 10.1002/ctm2.70324

**Published:** 2025-05-07

**Authors:** Xiaochen Wang, Yijie Su, Bei Lan, Xuanyuan Li, Bodi Zhang, Liang Zhang, Yingmei Wang, Chunze Zhang, Chenghao Xuan

**Affiliations:** ^1^ Key Laboratory of Breast Cancer Prevention and Therapy (Ministry of Education); The Province and Ministry Co‐sponsored Collaborative Innovation Center for Medical Epigenetics; Key Laboratory of Immune Microenvironment and Disease (Ministry of Education); Department of Biochemistry and Molecular Biology, School of Basic Medical Sciences, Tianjin Medical University Tianjin China; ^2^ Research Center of Translational Medicine Jinan Central Hospital Affiliated to Shandong First Medical University Jinan China; ^3^ Department of Gynecology and Obstetrics Tianjin Medical University General Hospital Tianjin China; ^4^ Department of Colorectal Surgery Tianjin Union Medical Center Tianjin China

**Keywords:** ferroptosis, hepatocellular carcinoma, proliferation, Sorafenib, USP22

## Abstract

**Background:**

Hepatocellular carcinoma remains one of the most lethal cancers, characterized by poor prognosis and low life expectancy. Unfortunately, there are very few molecular therapeutic options available for it. Sorafenib is a current standard first‐line treatment for advanced hepatocellular carcinoma, however, drug resistance significantly limits its therapeutic efficacy.

**Methods:**

Ubiquitin‐specific protease 22 (USP22) expression level and its prognostic significance in hepatocellular carcinoma were analyzed using The Cancer Genome Atlas (TCGA) database. A series of cellular experiments related to cell proliferation and ferroptosis, and mouse tumor‐bearing experiments were performed to investigate the role of USP22 in hepatocellular carcinoma cell growth and Sorafenib resistance. Flag affinity purification coupled with mass spectrometry, co‐immunoprecipitation, and ubiquitination assays were conducted to identify direct substrates of USP22. Spike‐in chromatin‐immunoprecipitation (ChIP)‐seq, RNA‐seq, and ChIP assays were employed to explore the transcriptional substrates of USP22 as an H2BK120ub deubiquitinase.

**Results:**

Analysis of TCGA database reveals that USP22 is highly expressed in hepatocellular carcinoma tissues, which is closely associated with poor patient prognosis. Our data further indicates that USP22 promotes the proliferation of hepatocellular carcinoma cells via deubiquitinating and stabilizing cyclin‐dependent kinase 11B (CDK11B). Additionally, USP22 acts as a novel inducer of Sorafenib resistance and suppresses Sorafenib‐triggered ferroptosis in hepatocellular carcinoma cells. It reduces the transcription of transferrin receptor (TFRC) by decreasing H2BK120ub occupancy at *TFRC* transcription start site (TSS) downstream region, thereby inhibiting ferroptosis upon Sorafenib treatment. Finally, animal experiments confirm the role of USP22 in promoting hepatocellular carcinoma cell growth and Sorafenib resistance in vivo. Taken together, this study demonstrates that USP22 promotes hepatocellular carcinoma growth and inhibits Sorafenib‐induced ferroptosis by deubiquitinating non‐histone substrate CDK11B and histone H2B, respectively.

**Conclusions:**

Our findings suggest USP22 as a promising prognostic biomarker and therapeutic target for hepatocellular carcinoma patients, particularly those with Sorafenib resistance.

**Key points:**

- USP22 promotes the proliferation of hepatocellular carcinoma cells by deubiquitinating and stabilizing cyclin‐dependent kinase CDK11B.
- USP22 enhances Sorafenib resistance of hepatocellular carcinoma cells by inhibiting ferroptosis through the USP22/H2BK120ub/TFRC axis.

## Graphical abstract

USP22 promotes the proliferation of hepatocellular carcinoma cells via deubiquitinating and stabilising cyclin‐dependent kinase CDK11B, and it inhibits Sorafenib‐induced ferroptosis by decreasing H2BK120ub occupancy at *TFRC* TSS downstream region.

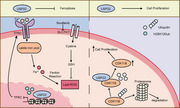

## 1 INTRODUCTION

According to GLOBOCAN data, liver cancer was the sixth most common cancer and the third leading cause of cancer‐related death worldwide, with 865,269 new cases and 757,948 deaths in 2022.^1^ Hepatocellular carcinoma is the predominant form of liver cancer, accounting for approximately 90% of cases.^2^ The 5‐year survival rate for hepatocellular carcinoma patients is less than 20%, which is largely due to the delayed diagnosis,^3^, ^4^ and the lack of effective targeted therapy options. Therefore, identifying key driving factors in the occurrence and development of hepatocellular carcinoma is crucial for its diagnosis and treatment.

For patients with advanced hepatocellular carcinoma, Sorafenib, a multi‐targeted tyrosine kinase inhibitor, is a first‐line systemic treatment drug.^5^ Unfortunately, Sorafenib provides only a modest survival benefit, with a median overall survival extension of approximately 3 months according to a multicentre, phase III, double‐blind clinical trial.^6^ Moreover, most patients develop acquired resistance within 6 months.^5^ Consequently, identifying genes involved in Sorafenib resistance is important for improving the therapeutic efficacy of Sorafenib for hepatocellular carcinoma patients.

Growing evidences demonstrate that Sorafenib exerts anti‐tumour effects in hepatocellular carcinoma via inducing ferroptosis.^7^, ^8^ Ferroptosis is an iron‐dependent, non‐apoptotic form of regulated cell death.^9^ It is executed through excessive lipid peroxidation, primarily mediated by the suppression of cystine‐glutamate antiporter system Xc^–^/glutathione (GSH)/glutathione peroxidase 4 (GPX4) axis, iron accumulation and the peroxidation of polyunsaturated fatty acids.^10^, ^11^ Sorafenib has been shown to induce ferroptosis by inhibiting the activity of system Xc^−^ and elevating intracellular iron levels.^12^ Facilitating ferroptosis may be a promising strategy to overcome Sorafenib resistance.

Ubiquitination is a key post‐translational modification of proteins that affects their stability, intracellular localisation and enzymatic activity.^13^ This process is reversible and can be reversed by deubiquitinases (DUBs), with ubiquitin‐specific peptidase 22 (USP22) being a prominent member of the largest subfamily of DUBs. USP22 can deubiquitylate both histone and non‐histone substrates. As a core component of the Spt‐Ada‐Gcn5 acetyltransferase (SAGA) chromatin modifying complex, USP22 catalyses the deubiquitination of histones H2A and H2B, thereby influencing gene transcription.^14^ As a member of an 11‐gene ‘death‐from‐cancer’ signature,^15^ USP22's non‐histone substrates play a critical role in mediating its pro‐oncogenic function. It has been reported that USP22 promotes cancer cell growth by deubiquitinating cyclin B1,^16^ proto‐oncogene MYC (c‐Myc)^17^ and far upstream element binding protein 1 (FBP1),^18^ while inhibiting apoptosis by deubiquitinating sirtuin 1 (Sirt1).^19^ However, there has been a lack of thorough investigation into the role of USP22 in hepatocellular carcinoma development and Sorafenib resistance.

In this study, we demonstrate that USP22 promotes the proliferation of hepatocellular carcinoma cells by stabilising cyclin‐dependent kinase 11B (CDK11B). Additionally, USP22 downregulates transferrin receptor (*TFRC*) transcription by removing H2B lysine 120 ubiquitination (H2BK120ub) from *TFRC* transcription start site (TSS) downstream region, thereby inhibiting Sorafenib‐induced ferroptosis in hepatocellular carcinoma. Our findings underscore the crucial role of USP22 in hepatocellular carcinoma development and Sorafenib resistance, suggesting that USP22 may serve as a promising therapeutic target for hepatocellular carcinoma.

## 2 RESULTS

### 2.1 USP22 promotes the proliferation of hepatocellular carcinoma cells

**FIGURE 1:**
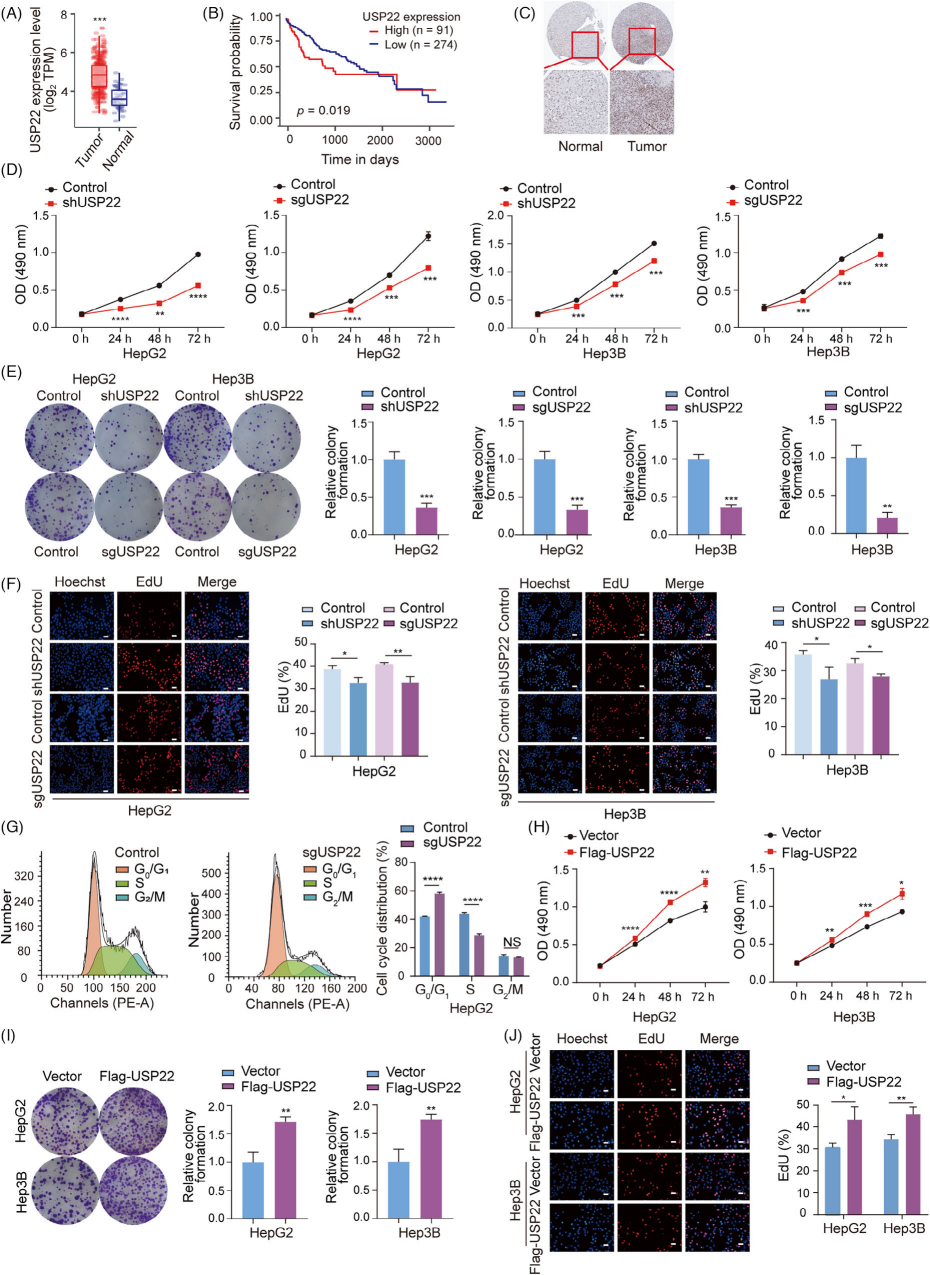
Ubiquitin‐specific protease 22 (USP22) promotes the proliferation of hepatocellular carcinoma cells. (A) Analysis of USP22 mRNA levels in 371 hepatocellular carcinoma samples and 50 normal liver tissues from the TCGA database using TIMER2.0 web platform (http://timer.cistrome.org/). ^***^ *p *< .001 (Wilcoxon test). (B) Kaplan‒Meier survival curve shows the overall survival of hepatocellular carcinoma patients with high or low USP22 expression analysed via UALCAN platform (https://ualcan.path.uab.edu/), *p* = .019 (log‐rank test). (C) Immunohistochemistry staining of USP22 in hepatocellular carcinoma tissue and normal liver tissue was obtained from The Human Protein Atlas (THPA) database. (D) 3‐(4,5‐Dimethylthiazol‐2‐yl)‐2,5‐diphenyl‐2H‐tetrazolium bromide (MTT) assays were performed in HepG2 and Hep3B cells with *USP22* knockout/knockdown or not. (E) HepG2 and Hep3B cells with *USP22* knockout/knockdown or control cells were cultured for 2 weeks and stained with crystal violet. Number of colonies was counted. Compared with the control group, the relative number of colonies is shown in a bar chart. (F) HepG2 and Hep3B cells with *USP22* knockout/knockdown or not were subjected to EdU‐incorporation assays, and the percentage of EdU‐positive cells was calculated. Scale bar: 50 µm. (G) Flow cytometry was used to analyse the cell cycle distribution of HepG2 cells with or without *USP22* knockout, and the proportion of cells in each phase was quantified. (H) MTT assays were conducted in HepG2 and Hep3B cells with or without *USP22* overexpression. (I) Colony formation assays were performed in HepG2 and Hep3B cells with *USP22* overexpression or not. (J) EdU‐incorporation assays were conducted in indicated cells. Scale bar: 50 µm. For figures (D‒J), data are mean ± SD for *n* = 3; NS: *p* > .05, ^*^ *p* < .05, ^**^ *p* < .01, ^***^ *p* < .001, ^****^ *p* < .0001 (Student's *t*‐test).

To investigate the role of USP22 in hepatocellular carcinoma development, we initially analysed USP22 expression levels in hepatocellular carcinoma tissues and normal tissues utilising the The Cancer Genome Atlas (TCGA) database. Our analysis unveiled a significant upregulation of *USP22* mRNA levels in hepatocellular carcinoma tissues compared to adjacent normal tissues (Figure 1A). Moreover, *USP22* mRNA expression levels were higher in hepatocellular carcinoma samples than in adjacent non‐tumour tissues in Chinese hepatocellular carcinoma patients using HCCDB6 dataset from the HCCDB v2.0 database^20^, ^21^, ^22^ (Figure S1A). Then, we delved into the correlation between USP22 expression level and patient prognosis, indicating that individuals with high *USP22* mRNA levels exhibited notably poorer prognosis (Figure 1B). Meanwhile, immunohistochemical staining data sourced from The Human Protein Atlas database corroborated a marked increase in USP22 protein expression in hepatocellular carcinoma tissues than normal tissues (Figure 1C). These series data suggested a potential involvement of USP22 in the progression of hepatocellular carcinoma.

To explore the impact of *USP22* knockout/knockdown on the proliferative capacity of hepatocellular carcinoma cells, we employed lentiviruses expressing either *USP22* single guide RNAs (sgRNAs) together with Cas9 or *USP22* shRNAs to infect HepG2, Hep3B and Huh7 cells, establishing cell populations stably expressing Cas9 and *USP22* sgRNA or *USP22* shRNA (Figure S1B). The efficiency of *USP22* knockout/knockdown was confirmed through Western blot analysis (Figure S1C). These *USP22* knockout/knockdown and control cells were subsequently utilised for experiments related to cell proliferation. First, we performed 3‐(4,5‐dimethylthiazol‐2‐yl)‐2,5‐diphenyl‐2H‐tetrazolium bromide (MTT) assays to assess cell viability. The results showed a significant decrease in cell viability following *USP22* knockout/knockdown in HepG2, Hep3B and Huh7 cells (Figures 1D and S2A). Additionally, colony formation ability of hepatocellular carcinoma cells was suppressed subsequent to *USP22* knockout/knockdown (Figures 1E and S2B). Furthermore, 5‐Ethynyl‐2'‐deoxyuridine (EdU)‐incorporation assays indicated that USP22 depletion reduced the percentage of cells in the S phase (Figures 1F and S2C). Cell cycle analysis by flow cytometry also showed increased percentage of cells in G_0_/G_1_ phase and decreased cells in S phase in *USP22* knockout groups (Figure 1G), suggesting the inhibition of cell proliferation.

To investigate the effect of *USP22* overexpression on hepatocellular carcinoma cell proliferation, we infected HepG2 and Hep3B cells with lentiviruses and established cell lines stably overexpressing Flag‐USP22 or not (Figure S1D). Subsequently, we performed MTT, colony formation and EdU‐incorporation assays. The results showed that *USP22* overexpression led to enhanced cell viability, increased colony formation ability and promoted proportion of EdU‐positive cells (Figure 1H‒J). Collectively, these findings demonstrated that USP22 promotes the proliferation of hepatocellular carcinoma cells in vitro.

### 2.2 USP22 interacts with CDK11B

**FIGURE 2:**
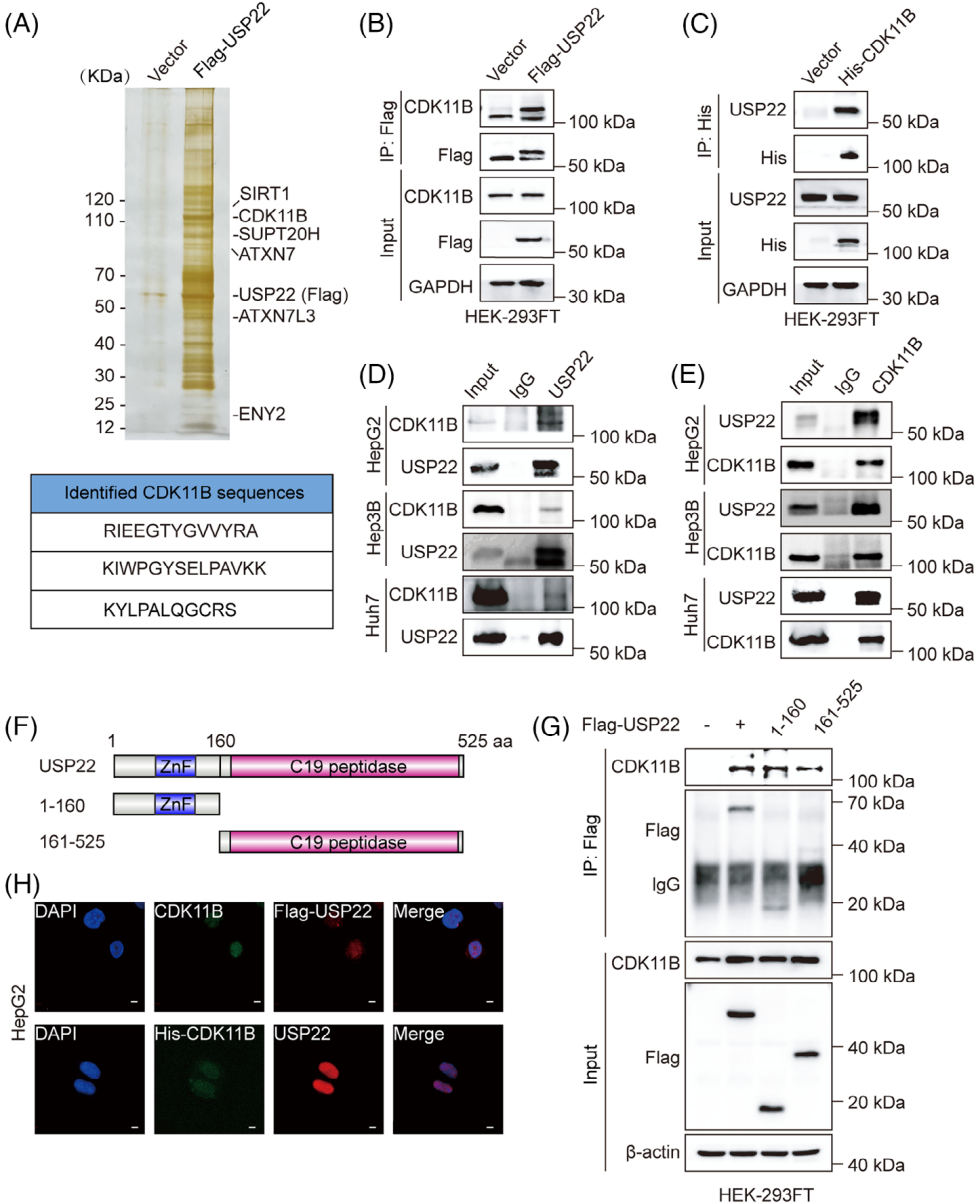
Ubiquitin‐specific protease 22 (USP22) interacts with cyclin‐dependent kinase 11B (CDK11B). (A) Cellular extracts from HEK‐293FT cells expressing Flag (vector) or Flag‐USP22 were immunopurified with anti‐Flag affinity columns and eluted with Flag peptides. The eluates were resolved by SDS‒PAGE, silver‐stained, and analysed by mass spectrometry. (B) Whole‐cell lysates from HEK‐293FT cells transfected with Flag‐USP22 or empty vectors were prepared and immunoprecipitation was performed with anti‐Flag followed by immunoblotting with antibodies against Flag and CDK11B. (C) Cell lysates from HEK‐293FT cells transfected with His‐CDK11B or empty vectors were prepared and immunoprecipitation was performed with anti‐His followed by Western blotting with anti‐His and anti‐USP22. (D) Immunoprecipitation assays were performed with anti‐USP22 followed by immunoblotting with anti‐CDK11B in HepG2, Hep3B and Huh7 cells. (E) Immunoprecipitation assays were performed with anti‐CDK11B followed by immunoblotting with anti‐USP22 in HepG2, Hep3B and Huh7 cells. (F) Schematic diagram of USP22 full‐length and truncations. (G) Whole‐cell lysates from HEK‐293FT cells transfected with empty vector, Flag‐USP22, Flag‐USP22 (1‒160) or Flag‐USP22 (161‒525) expression constructs were prepared, and immunoprecipitation was performed with anti‐Flag, followed by immunoblotting with indicated antibodies. (H) The distribution of Flag‐USP22 and endogenous CDK11B, or GFP‐CDK11B and endogenous USP22 was detected by immunofluorescence assays using anti‐Flag with anti‐CDK11B, or anti‐USP22 in HepG2 cells. 4′,6‐diamidino‐2‐phenylindole (DAPI) staining was conducted to visualise the cell nuclei. Scale bar: 5 µm.

To elucidate the mechanisms underlying USP22‐promoted proliferation of hepatocellular carcinoma cells, we speculated that the deubiquitination of substrate proteins catalysed by USP22 probably accounted for the regulatory effects we observed. To test this, we applied Flag affinity purification and mass spectrometry to identify proteins that potentially interact with USP22 in vivo. The lysates of HEK‐293FT cells expressing Flag‐USP22 were prepared and subjected to Flag affinity purification. The eluate was resolved on Sodium Dodecyl Sulfate PolyAcrylamide Gel Electrophoresis (SDS‐PAGE) and silver‐stained (Figure 2A). Mass spectrometric analysis of the resolved protein bands showed that besides components of the SAGA complex, including ATXN7, ENY2, ATXN7L3 and SUPT20H,^23^ as well as SIRT1,^19^ which were all previously reported to interact with USP22, CDK11B, a member of the cyclin‐dependent kinases (CDKs), was also co‐purified with USP22 (Figure 2A).

To validate affinity purification results, exogenous co‐immunoprecipitation (IP) was first performed. Total proteins from HEK‐293FT cells expressing Flag‐USP22 or control vectors were first immunoprecipitated with anti‐Flag, followed by immunoblotting with antibodies against CDK11B. The results showed that Flag‐USP22 indeed interacted with CDK11B (Figure 2B). On the other hand, total proteins from HEK‐293FT cells expressing His‐CDK11B or control vectors were immunoprecipitated with anti‐His, followed by Western blotting with anti‐USP22, also demonstrating the interaction between His‐CDK11B and USP22 (Figure 2C). Furthermore, total proteins from HepG2, Hep3B and Huh7 cells were respectively extracted and subjected to co‐IP using antibodies against endogenous proteins. The results manifested that USP22 interacts with CDK11B in hepatocellular carcinoma cells (Figure 2D,E).

USP22 protein contains an N‐terminal zinc‐finger (ZnF) and a C19 ubiquitin‐specific peptidase (C19 peptidase) domain.^24^, ^25^ To illustrate the molecular detail involved, co‐IP assays were performed in HEK‐293FT cells expressing Flag (vector), Flag‐USP22, Flag‐USP22 (1‒160 aa) or Flag‐USP22 (161‒525 aa) using anti‐Flag. The results showed that USP22 as well as its ZnF domain (1‒160 aa) and C19 peptidase (161‒525 aa) were all able to interact with CDK11B (Figure 2F,G). Moreover, immunofluorescent staining was conducted, revealing the co‐localisation of USP22 and CDK11B in hepatocellular carcinoma cells (Figures 2H and S3). Taken together, these results indicated that USP22 interacts with CDK11B in hepatocellular carcinoma cells.

### 2.3 USP22 catalyses the deubiquitination of CDK11B

**FIGURE 3:**
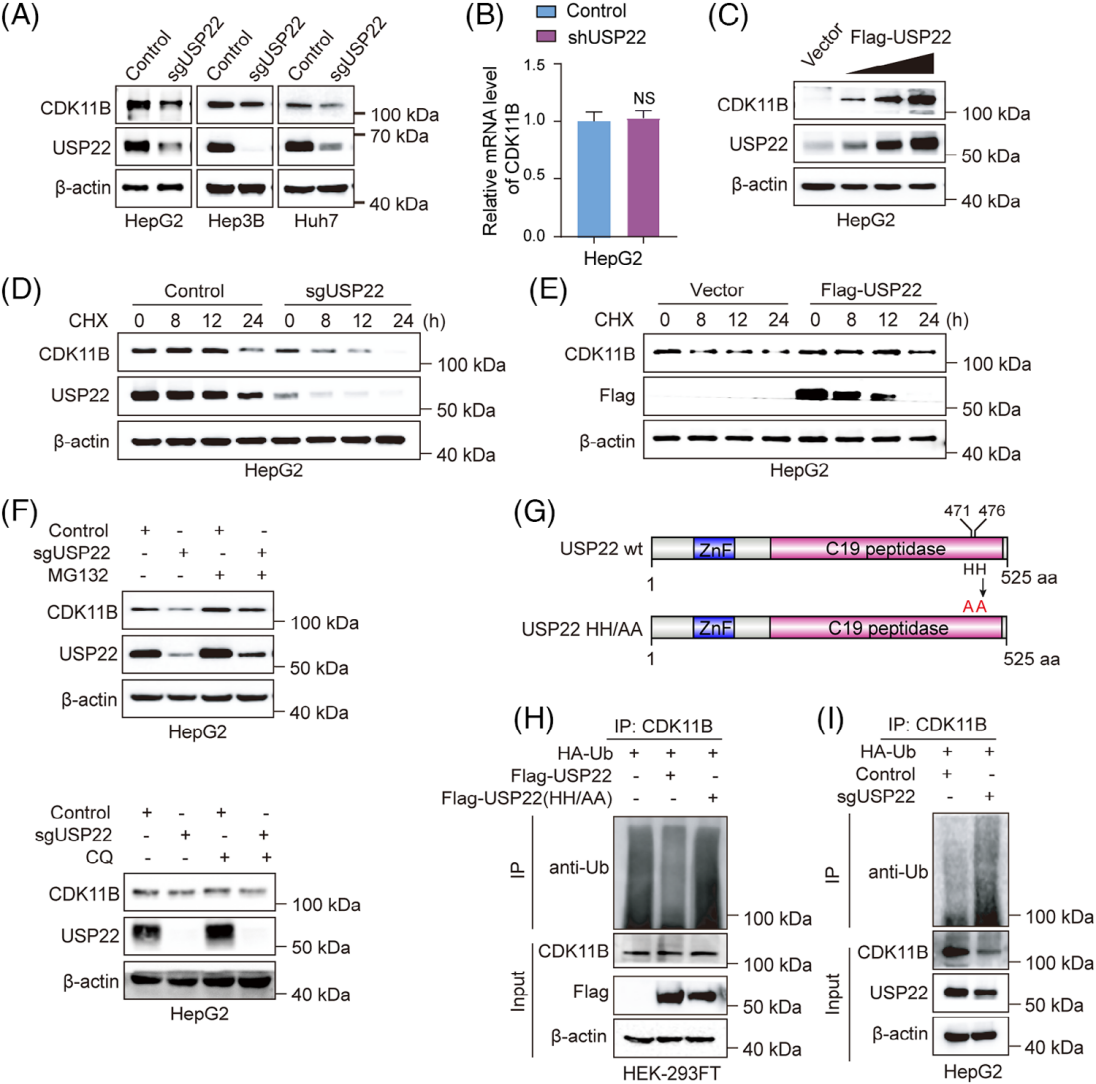
Ubiquitin‐specific protease 22 (USP22) catalyses the deubiquitination of cyclin‐dependent kinase 11B (CDK11B). (A) Western blotting was used to detect the protein level of CDK11B in control and USP22 knockout cells (HepG2/Hep3B/Huh7). (B) The mRNA level of *CDK11B* in control and USP22 knockdown HepG2 cells was detected by quantitative real‐time RT‐PCR assays. Data are mean ± SD for *n* = 3; NS: *p* > .05 (Student's *t*‐test). (C) Empty vectors, 0.5, 1 or 2 µg Flag‐USP22 expression plasmids were transfected into HepG2 cells, and the protein levels of USP22 and CDK11B were detected by Western blotting. (D) Half‐lives of CDK11B protein were examined in control and *USP22* knockout cells using cycloheximide (CHX) pulse‐chase assays. (E) Half‐lives of CDK11B protein were examined in control and *USP22* overexpressing cells using CHX pulse‐chase assays. (F) Protein level of CDK11B was detected by Western blotting in control and *USP22* knockout HepG2 cells treated with dimethyl sulphoxide (DMSO), MG132 (10 µM) or chloroquine (CQ, 20 µM) for 4 h. (G) Schematic diagram of mutation sites in USP22 enzymatic mutant. (H) HEK‐293FT cells were transfected with HA‐ubiquitin expression plasmids together with Flag‐USP22, Flag‐USP22 enzymatic mutant or control plasmids. Forty‐eight hours post‐transfection, the cells were treated with MG132 (10 µM) for 4 h and then the ubiquitination of CDK11B was detected by Western blotting using anti‐Ub after immunoprecipitation by anti‐CDK11B. (I) HA‐ubiquitin expression plasmids were transfected into *USP22* knockout HepG2 cells and control cells. Forty‐eight hours post‐transfection, the cells were treated with MG132 (10 µM) for 4 h and the ubiquitination of CDK11B was detected by Western blotting using anti‐Ub after immunoprecipitation by anti‐CDK11B.

USP22 functions as an ubiquitin hydrolase, stabilising target proteins through the removal of ubiquitin molecules.^26^ The observed interaction between USP22 and CDK11B raised the question that whether USP22 catalyses the deubiquitination of CDK11B protein. To validate this hypothesis, we initially examined CDK11B mRNA and protein expression changes following *USP22* knockout/knockdown. Our results demonstrated a decrease in CDK11B protein level upon *USP22* knockout/knockdown (Figure 3A), while *CDK11B* mRNA level remained unchanged (Figure 3B). Consistently, CDK11B protein level was promoted by the overexpression of USP22 in a dose‐dependent manner (Figure 3C). These results indicated that USP22 does not regulate the transcription of *CDK11B*, but its level has positive correlation with CDK11B protein level.

Then, we performed cycloheximide (CHX) pulse‐chase assay to examine the effects of *USP22* knockout and overexpression on the half‐life of CDK11B protein. The results indicated that the half‐life of CDK11B protein was significantly shortened after *USP22* knockout (Figure 3D), whereas overexpression of *USP22* led to an extension of CDK11B half‐life (Figure 3E). To further determine the degradation pathway of CDK11B, we employed MG132 and chloroquine to inhibit ubiquitin‒proteasome and lysosome pathways, respectively. The decreased protein level of CDK11B in *USP22* knockout HepG2 cells was rescued by MG132 but not by chloroquine, demonstrating that *USP22* knockout leads to CDK11B degradation via the ubiquitin‒proteasome pathway (Figure 3F). We further investigated whether USP22 targets CDK11B for deubiquitination. The plasmids encoding Flag‐USP22 wild‐type or catalytically inactive Flag‐USP22^HH/AA^ (Figure 3G) were co‐transfected with HA‐ubiquitin into HEK‐293FT cells. As expected, ectopic expression of wild‐type USP22 but not USP22^HH/AA^ (Figure 3G) reduced the poly‐ubiquitination of CDK11B (Figure 3H), while *USP22* knockout augmented the poly‐ubiquitination of CDK11B (Figure 3I). Collectively, these data strongly indicated the notion that USP22 stabilises CDK11B protein via direct deubiquitination.

### 2.4 USP22 facilitates the proliferation of hepatocellular carcinoma cells by stabilising CDK11B protein

**FIGURE 4:**
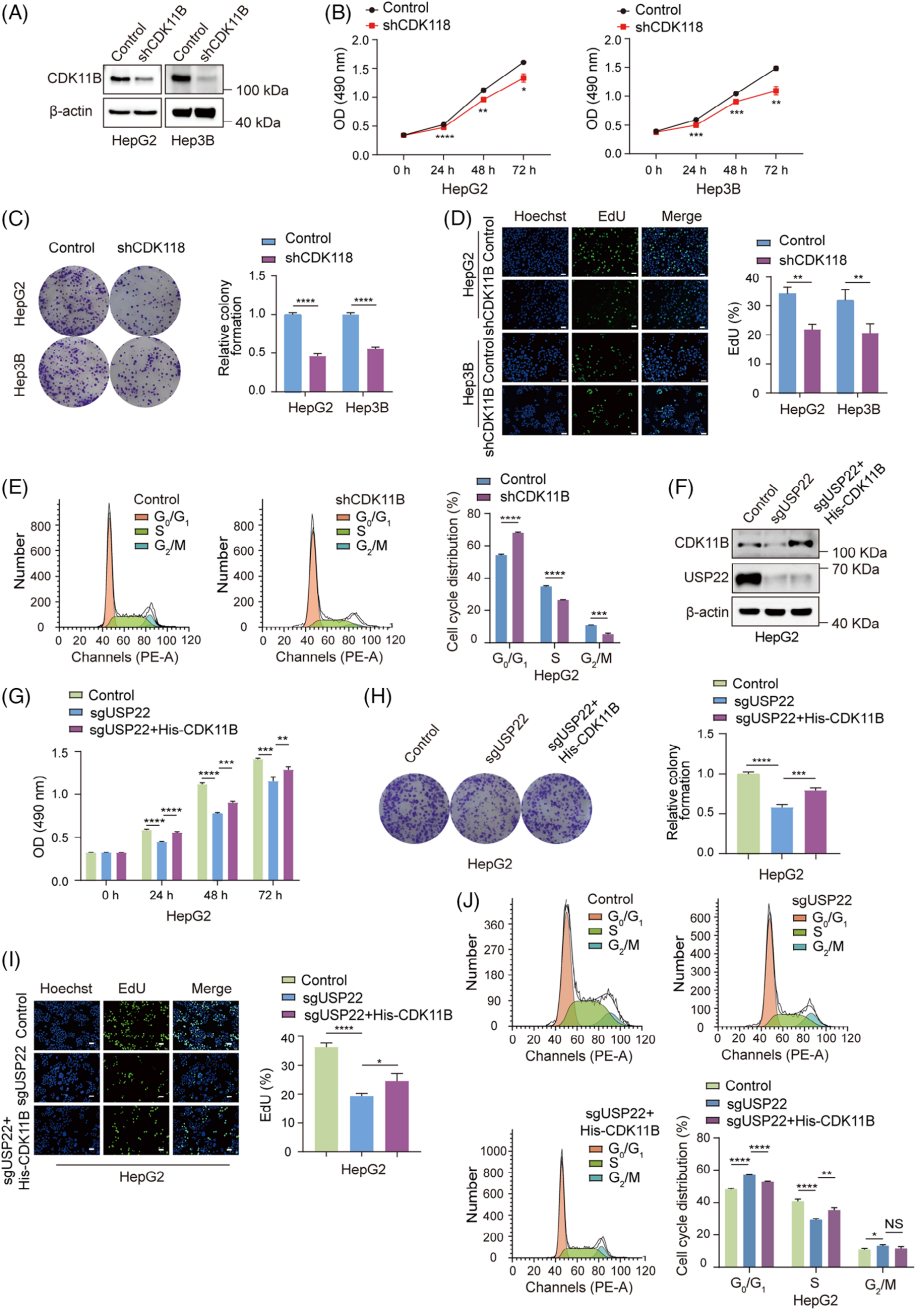
Ubiquitin‐specific protease 22 (USP22) promotes hepatocellular carcinoma cell proliferation through stabilising cyclin‐dependent kinase 11B (CDK11B) protein. (A) Western blotting was used to detect the protein level of CDK11B in HepG2 cells expressing control of *CDK11B* shRNAs. (B) 3‐(4,5‐Dimethylthiazol‐2‐yl)‐2,5‐diphenyl‐2H‐tetrazolium bromide (MTT) assays were performed in HepG2 and Hep3B cells expressing control or *CDK11B* shRNAs. (C) Colony formation assays were conducted in HepG2 and Hep3B cells expressing control or *CDK11B* shRNAs. Number of colonies was counted. (D) EdU‐incorporation assays were performed in indicated cells, and the percentage of EdU‐positive cells was calculated. Scale bar: 50 µm. (E) Cell cycle distribution of HepG2 cells expressing control or *CDK11B* shRNAs was analysed by flow cytometry, and the percentage of cells in each phase was quantified. For figures (B‒E), data are mean ± SD for *n* = 3; ^*^ *p *< .05, ^**^ *p *< .01, ^***^ *p *< .001, ^****^ *p *< .0001 (Student's *t*‐test). (F) Western blotting was used to detect the protein levels of USP22 and CDK11B in control cells and *USP22* knockout HepG2 cells transfected with His‐CDK11B expression construct or not. (G) MTT assays were performed in the indicated cells. (H) Colony formation assays were conducted in the indicated cells. (I) EdU‐incorporation assays were performed in the indicated cells. Scale bar: 50 µm. (J) Flow cytometry analyses were performed to assess the cell cycle distribution in the indicated cells, with the percentage of cells in each phase quantified. For figures (G‒J), data are mean ± SD for *n* = 3; NS: *p* > .05, ^*^ *p *< .05, ^**^ *p *< .01, ^***^ *p *< .001, ^****^ *p *< .0001 (one‐way ANOVA followed by Tukey's test for multiple comparisons).

CDKs are a group of serine/threonine kinases involved in the regulation of cell cycle progression.^27^ Recent studies have also highlighted the essential role of CDK11 in the progression of various cancers, including osteosarcoma,^28^ liposarcoma^29^ and breast cancer.^30^ Notably, *CDK11* knockdown has been shown to induce cell cycle arrest at the G_1_ phase in both breast cancer^30^ and melanoma cells.^31^ To establish the relationship between the promotion of cell proliferation and the stabilisation of CDK11B protein caused by *USP22* overexpression, we intended to first examine the effects of *CDK11B* knockdown on hepatocellular carcinoma cell proliferation. The efficiency of *CDK11B* knockdown by its specific shRNAs in HepG2 and Hep3B cells was confirmed through Western blot analysis (Figure 4A). Subsequent MTT and colony formation assays demonstrated a significantly suppressed cell viability and colony formation ability upon *CDK11B* knockdown in HepG2 and Hep3B cells (Figure 4B,C). EdU‐incorporation assays revealed suppressed cell proliferation following *CDK11B* knockdown (Figure 4D). Additionally, flow cytometry analyses showed that *CDK11B* knockdown led to an increase in the percentage of G_0_/G_1_ phase cells and a decrease in S phase cells (Figure 4E). To further validate that USP22 increases hepatocellular carcinoma cell proliferation by stabilising CDK11B protein, we overexpressed His‐CDK11B in *USP22* knockout cells (Figure 4F) and evaluated cell proliferation. The results of MTT, colony formation, EdU‐incorporation and flow cytometry assays demonstrated that overexpression of *CDK11B* significantly reversed the reduction in cell proliferation induced by *USP22* knockout (Figure 4G‒J). These findings collectively indicated that *USP22* knockout inhibits the proliferation of hepatocellular carcinoma cells through destabilising CDK11B protein.

### 2.5 USP22 inhibits Sorafenib‐induced ferroptosis in hepatocellular carcinoma cells

**FIGURE 5:**
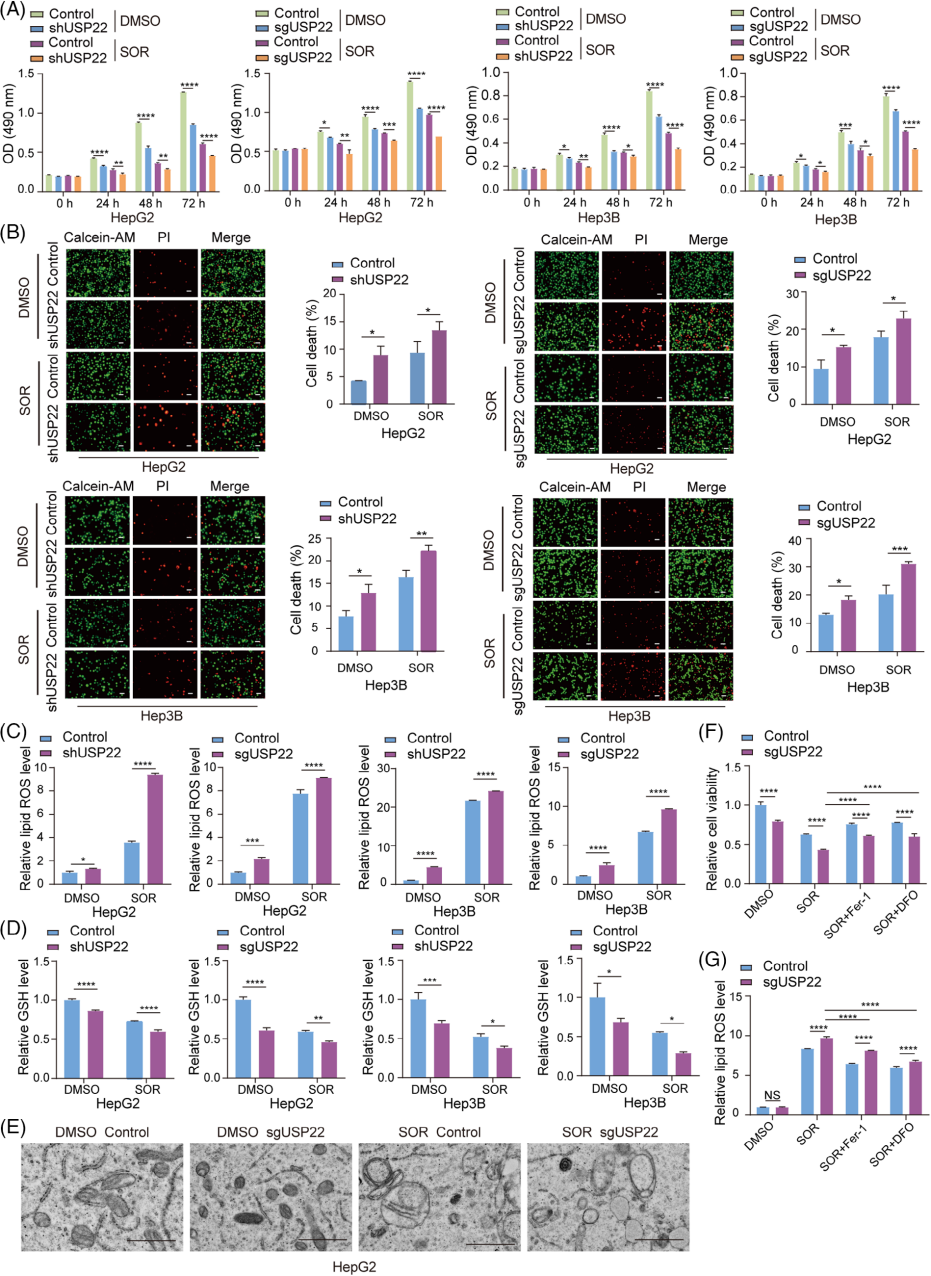
Ubiquitin‐specific protease 22 (USP22) inhibits Sorafenib‐induced ferroptosis in hepatocellular carcinoma cells. (A) Control or *USP22* knockout/knockdown HepG2 and Hep3B cells were treated with dimethyl sulphoxide (DMSO) or 10 µM Sorafenib for indicated times, and then cell viability was examined using 3‐(4,5‐dimethylthiazol‐2‐yl)‐2,5‐diphenyl‐2H‐tetrazolium bromide (MTT) assays. (B) Control or *USP22* knockout/knockdown HepG2 and Hep3B cells were treated with DMSO or 10 µM Sorafenib for 24 h, then stained with Calcein‐AM/propidium iodide (PI), and observed under a fluorescent microscope (left panel). Percentage of dead cells was calculated (right panel). Scale bar: 50 µm. (C) Control or *USP22* knockout/knockdown HepG2 and Hep3B cells were treated with DMSO or 10 µM Sorafenib for 24 h, and cellular lipid reactive oxygen species (ROS) was detected. (D) Control or *USP22* knockout/knockdown HepG2 and Hep3B cells were treated with DMSO or 10 µM Sorafenib for 24 h, and cellular glutathione (GSH) was examined. (E) Morphological changes of mitochondria in control or *USP22* knockout HepG2 cells treated with DMSO or Sorafenib (10 µM, 24 h) were observed under a transmission electron microscope. Scale bar: 1 µm. (F) Control or *USP22* knockout HepG2 cells were treated with DMSO, Sorafenib (10 µM) or Sorafenib together with Fer‐1 (5 µM) or DFO (3 µM) for 24 h, and cell viability was detected using MTT assays. (G) Control or *USP22* knockout HepG2 cells were treated with indicated drugs for 24 h, and cellular lipid ROS was detected. For figures (A‒G), data are mean ± SD for *n* = 3; NS: *p *> .05, ^*^ *p *< .05, ^**^ *p *< .01, ^***^ *p *< .001, ^****^ *p *< .0001 (two‐way ANOVA followed by Tukey's test for multiple comparisons).

Sorafenib is a first‐line drug for advanced hepatocellular carcinoma treatment.^32^ However, resistance to Sorafenib is a common issue among hepatocellular carcinoma patients, limiting its effectiveness. USP22 was reported to be upregulated by Sorafenib treatment,^33^ suggesting its potential in modulating Sorafenib resistance. After we had confirmed that USP22 promotes the growth of hepatocellular carcinoma, we further focused on exploring whether USP22 regulates Sorafenib resistance in hepatocellular carcinoma. To test this hypothesis, HepG2 and Hep3B cells stably expressing USP22 sgRNA together with Cas9, or USP22 shRNA were treated with (DMSO) or Sorafenib, and then MTT assays were performed. The results revealed that *USP22* knockout/knockdown significantly inhibited cell growth in the presence of Sorafenib (Figure 5A). To further investigate the effect of *USP22* knockout/knockdown on Sorafenib‐induced cell death, Calcein‐AM/propidium iodide (PI) double staining assay was performed, showing that *USP22* knockout/knockdown increased the percentage of dead cells upon Sorafenib treatment (Figure 5B). These results illustrated that reduced expression of USP22 in hepatocellular carcinoma cells enhanced cell sensitivity to Sorafenib.

Since growing evidences demonstrate that Sorafenib exerts anti‐tumour effects in hepatocellular carcinoma via inducing ferroptosis,^7^, ^8^ we therefore examined several markers of ferroptosis in control and *USP22* knockout/knockdown cells to determine whether *USP22* knockout/knockdown affects Sorafenib‐induced ferroptosis. Ferroptosis, driven by iron‐dependent phospholipid peroxidation, is accompanied by increase in lipid reactive oxygen species (ROS) and reduction in cellular GSH.^10^, ^34^ Morphologically, ferroptotic cells exhibit condensed mitochondrial membrane densities, reduced mitochondrial volume, diminished mitochondrial cristae and ruptured outer membrane.^35^ Our results demonstrated that *USP22* knockout/knockdown increased cellular lipid ROS levels (Figure 5C), decreased cellular GSH levels (Figure 5D) and reduced mitochondrial cristae (Figure 5E) under Sorafenib treatment. Furthermore, the inhibited cell viability and the increased lipid ROS induced by *USP22* knockout/knockdown in the presence of Sorafenib can be rescued by the inhibitor of ferroptosis, Ferrostatin‐1 (Fer‐1) and 1,8‐Diazafluoren‐9‐one (DFO) (Figure 5F,G), further demonstrating that USP22 inhibition promotes Sorafenib‐induced ferroptosis in hepatocellular carcinoma cells.

Conversely, *USP22* overexpression inhibited ferroptosis induced by Sorafenib, as indicated by increased cell viability (Figure S4A), decreased cell death (Figure S4B), reduced cellular lipid ROS levels (Figure S4C) and elevated cellular GSH levels (Figure S4D).

Erastin, the first discovered inducer of ferroptosis, inhibits the activity of System Xc.^36^ RSL3, a GPX4 inhibitor, induces ferroptosis mainly by reducing GPX4 activity in cells.^37^ To further elucidate the role of USP22 in inhibiting ferroptosis, we treated HepG2 cells with *USP22* knockout and control cells with Sorafenib, Erastin and RSL3, respectively. We then measured cell viability and lipid ROS. Our results showed that *USP22* knockout enhanced ferroptosis induced by different drugs, as evidenced by decreased cell viability (Figure S5A) and elevated cellular lipid ROS levels (Figure S5B) compared to control cells. These findings collectively demonstrated that USP22 negatively regulates ferroptosis in hepatocellular carcinoma cells.

### 2.6 USP22 modulates cellular H2BK120ub level and the transcription of *TFRC*

**FIGURE 6:**
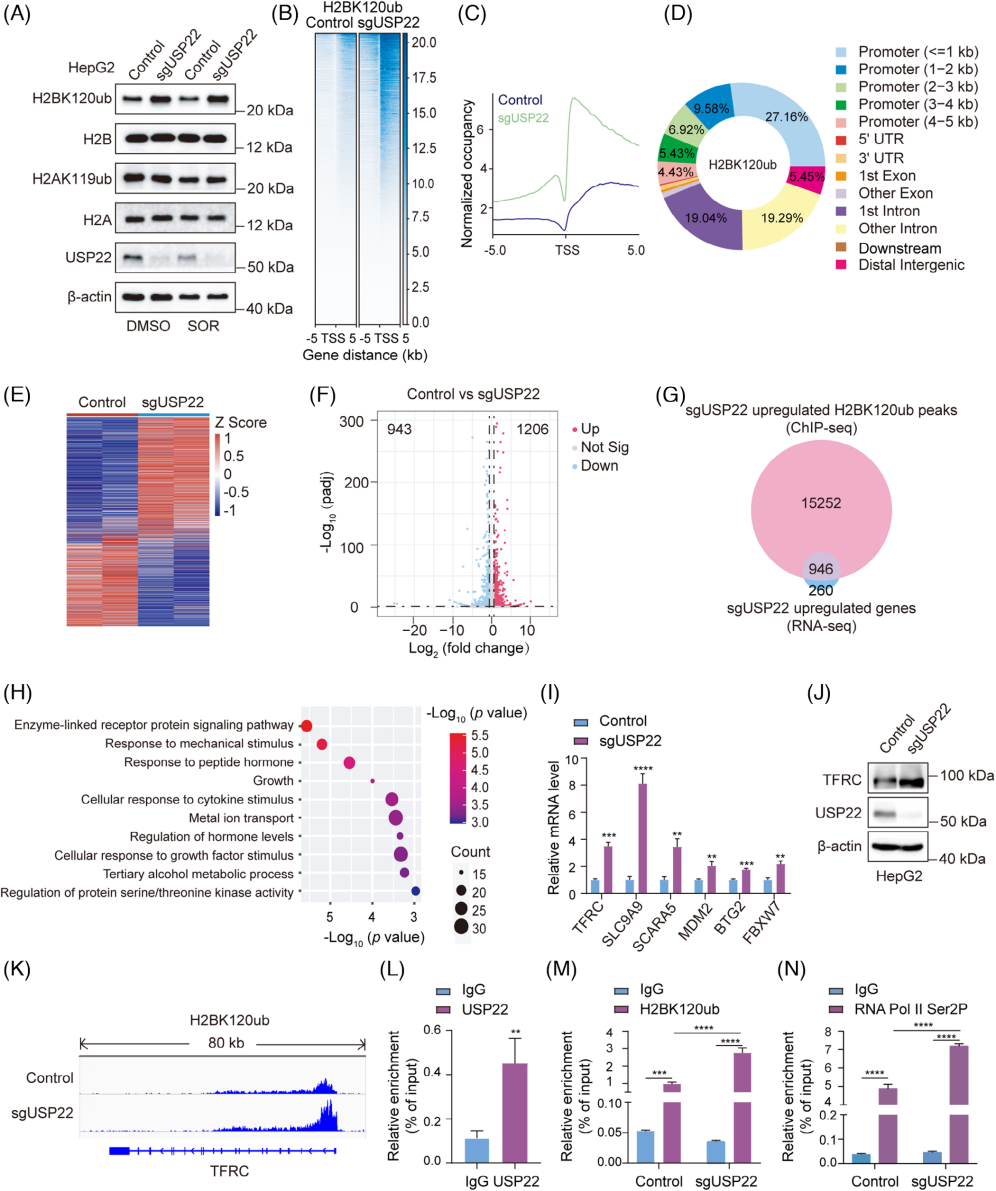
Ubiquitin‐specific protease 22 (USP22) modulates cellular H2BK120ub level and the transcription of transferrin receptor (TFRC). (A) Control or *USP22* knockout HepG2 cells were treated with dimethyl sulphoxide (DMSO) or 10 µM Sorafenib for 24 h, then cell lysate was subjected to Western blotting using indicated antibodies. (B and C) Spike‐in chromatin‐immunoprecipitation sequencing (ChIP‐seq) was performed in control or *USP22* knockout HepG2 cells treated with Sorafenib (10 µM, 24 h) using anti‐H2BK120ub. Heatmap (B) and profile diagram (C) show H2BK120ub signal levels and patterns across 5 kb upstream to 5 kb downstream of transcription start sites (TSSs). (D) Pie chart visualises the genomic distribution of H2AK120ub peaks upregulated by *USP22* knockout. The promoter (≤ 1 kb) represents both the upstream and downstream 1 kb range of TSSs. (E) The mRNAs from Sorafenib‐treated HepG2 cells stably expressing control or *USP22* sgRNAs together with Cas9 were extracted and subjected to RNA sequencing (RNA‐seq). Heatmaps show differentially expressed genes following *USP22* knockout. (F) Volcano plot shows the differentially expressed genes between HepG2 cells expressing control or *USP22* sgRNAs. Genes meeting the criteria of padj < .05 and fold change ≥ 1.5 are depicted as red dots for upregulated genes and blue dots for downregulated genes. (G) Venn diagram illustrates the overlap between genes with enhanced H2AK120ub peak signals and those with upregulated mRNA level mediated by *USP22* knockout, based on the integrated analysis of Spike‐in ChIP‐seq and RNA‐seq. (H) Gene Ontology (GO) pathway enrichment analysis of the overlapped gene set in (G). (I) Quantitative real‐time RT‐PCR assays were performed in control and *USP22* knockout HepG2 cells treated by Sorafenib (10 µM, 24 h). Data are mean ± SD for *n* = 3; ^**^ *p *< .01, ^***^ *p *< .001, ^****^ *p *< .0001 (Student's *t‐*test). (J) Western blotting was applied to detect the protein level of TFRC after *USP22* knockout in HepG2 cells upon Sorafenib treatment (10 µM, 24 h). (K) Genome browser view of the H2BK120ub density on *TFRC* gene obtained by Spike‐in ChIP‐seq in control and USP22 knockout HepG2 cells. (L) ChIP assays were performed in HepG2 cells using anti‐USP22 or immunoglobulin G (IgG), and then qPCR assays were executed with primer pairs targeting the TSS downstream of *TFRC*. Data are mean ± SD for *n* = 3; ^**^ *p *< .01 (Student's *t*‐test). (M and N) ChIP assays were performed in control and *USP22* knockout HepG2 cells using anti‐H2BK120ub, anti‐RNA Pol II Ser2P or IgG, and then qPCR assays were used to detect their enrichment with primer pairs targeting the TSS downstream of *TFRC*. Data are mean ± SD for *n* = 3; ^***^ *p *< .001, ^****^ *p *< .0001 (two‐way ANOVA followed by Tukey's test for multiple comparisons).

As a key subunit of SAGA complex, USP22 catalyses the removal of the mono‐ubiquitin moiety from both lysine 120 of H2B (H2BK120ub1) and lysine 119 of H2A (H2AK119ub1).^14^, ^38^ To detect whether USP22 participates in the deubiquitination of H2AK119ub and H2BK120ub during Sorafenib‐induced ferroptosis, we initially conducted Western blotting to assess the levels of H2AK119ub and H2BK120ub in *USP22* knockout or control cells treated with DMSO or Sorafenib. The results unveiled a significant elevation in the H2BK120ub level upon *USP22* knockout, which was further augmented after Sorafenib treatment, whereas the level of H2AK119ub remained largely unchanged (Figure 6A). To further delve into the impact of *USP22* knockout on the genome‐wide distribution of H2BK120ub, Spike‐in chromatin‐immunoprecipitation sequencing (ChIP‐seq) was performed using anti‐H2BK120ub in HepG2 cells with *USP22* knockout or not upon Sorafenib treatment. The results indicated a remarkable increase in the intensity of H2BK120ub, which is mainly located in coding regions immediately downstream of TSSs and within promoters (Figure 6B‒D).

To investigate the effect of *USP22* knockout on transcriptome, RNA sequencing (RNA‐seq) was conducted in *USP22* knockout HepG2 cells and control cells both with Sorafenib treatment, revealing that 1206 genes were upregulated and 943 genes were downregulated by *USP22* knockout (Figure 6E,F). As H2BK120ub modification is associated with transcriptional active genes, we performed a combined analyses of Spike‐in ChIP‐seq and RNA‐seq data. The analyses indicated that, in *USP22* knockout cells, there were a total of 16 198 genes with increased H2BK120ub peak signals, of which 946 genes had increased mRNA levels (Figure 6G). Gene Ontology pathway enrichment analysis of these 946 genes underscored their marked involvement in ‘metal ion transport’, which plays critical roles in ferroptosis (Figure 6H). Subsequently, the mRNA levels of ferroptosis‐related genes, including *TFRC*, *SCL9A9*, *SCARA5*, *MDM2*, *BTG2* and *FBXW7*, were detected using quantitative real‐time RT‐PCR (RT‐qPCR) experiments in control and *USP22* knockout cells. The results consistently corroborated the RNA‐seq findings, showing upregulation of the transcription of these genes in *USP22* knockout cells upon Sorafenib treatment (Figure 6I).

TFRC is a critical receptor responsible for importing iron from the extracellular environment into cells and was identified as a specific ferroptosis marker.^39^, ^40^ It was speculated that TFRC was essential for USP22‐mediated resistance to Sorafenib‐induced ferroptosis. The increase in TFRC protein level upon *USP22* knockout was further confirmed by Western blot analysis (Figure 6J). Our Spike‐in ChIP‐seq data presented by IGV also revealed an increased H2BK210ub occupancy on the TSS downstream of *TFRC* after USP22 depletion (Figure 6K). Furthermore, to detect whether USP22 directly binds to the TSS downstream of *TFRC*, ChIP‐qPCR assays were performed using USP22 antibodies in HepG2 cells. The results validated the direct binding of USP22 on the TSS downstream of *TFRC* (Figure 6L). Moreover, USP22 depletion increased the enrichment of H2BK120ub on the TSS downstream region of *TFRC* (Figure 6M). Additionally, H2BK120ub has been shown to facilitate transcription elongation,^41^ which is characterised by increased phosphorylation of serine 2 in the RNA polymerase II (RNA pol II) C‐terminal domain.^42^, ^43^ ChIP‐qPCR assay results further revealed enhanced enrichment of RNA Pol II Ser2P at TSS downstream region of *TFRC* in *USP22* knockout cells compared to control cells (Figure 6N). In summary, these findings suggested that *USP22* knockout enhances the occupation of H2BK120ub on the TSS downstream region of *TFRC*, thereby promoting *TFRC* transcription.

### 2.7 USP22 inhibits Sorafenib‐induced ferroptosis through downregulating *TFRC* transcription

**FIGURE 7:**
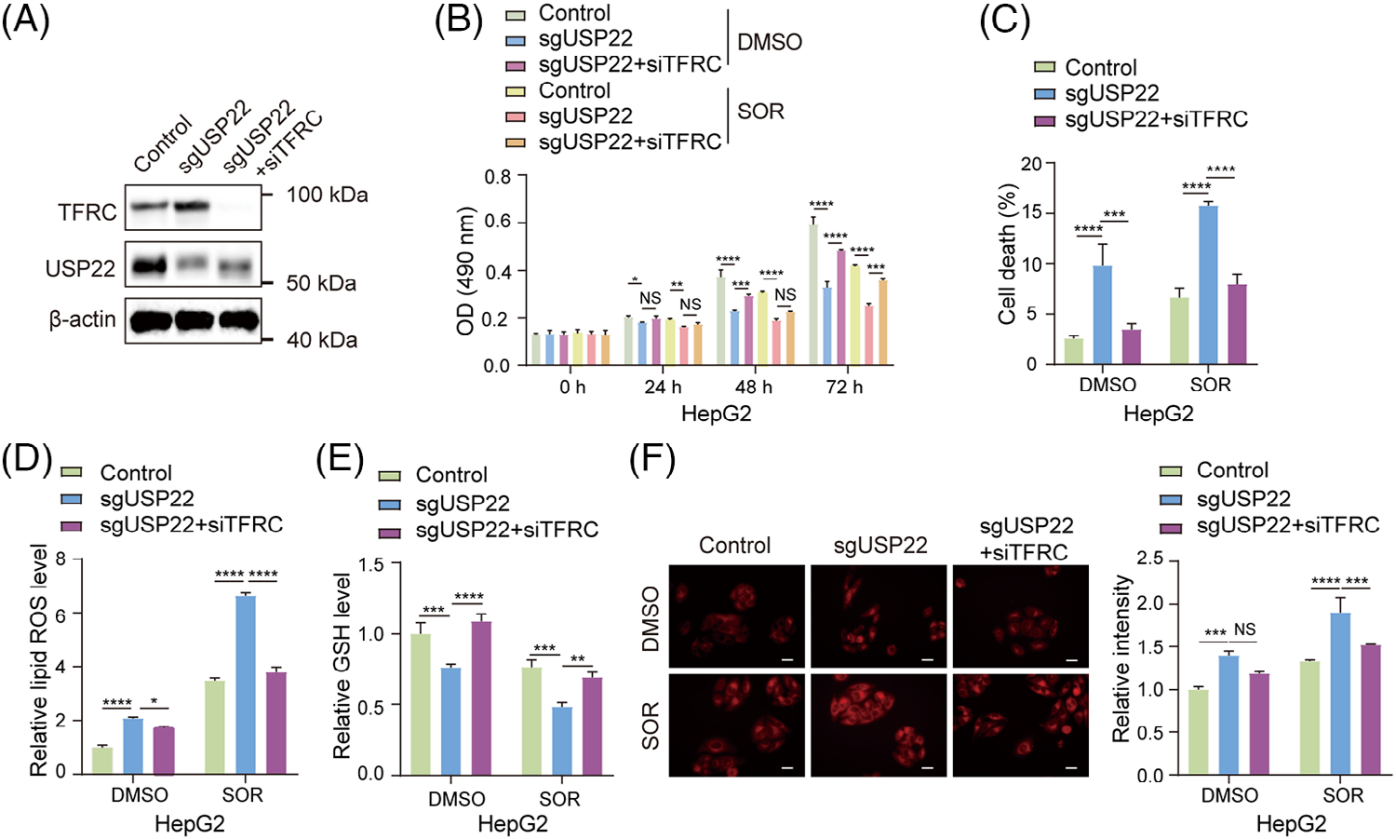
Ubiquitin‐specific protease 22 (USP22) inhibits Sorafenib‐induced ferroptosis through downregulating transferrin receptor (TFRC) transcription. (A) Western blot analysis of the protein levels of TFRC, USP22 and β‐actin in indicated cells. (B) Viability of indicated cells was measured using 3‐(4,5‐dimethylthiazol‐2‐yl)‐2,5‐diphenyl‐2H‐tetrazolium bromide (MTT) assays. (C) Calcein‐AM/propidium iodide (PI) staining was performed in indicated cells. Percentage of dead cells was calculated. (D) Cellular lipid ROS was detected in indicated cells. (E) Reduced glutathione (GSH) levels were detected after dimethyl sulphoxide (DMSO) or Sorafenib (10 µM) treatment for 24 h in HepG2 sgUSP22, sgUSP22 + siTFRC and control cells. (F) Intracellular Fe^2+^ was detected with the FerroOrange probe under a fluorescent microscope (red, FerroOrange‐stained Fe^2+^). Fe^2+^ fluorescence intensity was quantified by ImageJ. Scale bar: 5 µm. For figures (B‒F), data are mean ± SD for *n* = 3; NS: *p* > .05, ^*^ *p *< .05, ^**^ *p *< .01, ^***^ *p *< .001, ^****^ *p *< .0001 (two‐way ANOVA followed by Tukey's test for multiple comparisons).

To prove that the upregulation of *TFRC* transcription mediates the promotion of ferroptosis caused by *USP22* knockout, we knocked down *TFRC* expression through transfecting its specific small interfering RNAs (siRNAs) into *USP22* knockout cells (Figure 7A), and subsequently measured cell viability, cell death, cellular lipid ROS, cellular GSH and Fe^2+^ levels. The results revealed that knockdown of *TFRC* rescued the cell phenotype caused by *USP22* knockout, leading to the inhibition of Sorafenib‐induced ferroptosis, as evidenced by increased cell viability (Figure 7B), decreased percentages of cell death (Figure 7C), decreased cellular lipid ROS level (Figure 7D), promoted GSH level (Figure 7E) and reduced Fe^2+^ level (Figure 7F) in USP22‐depleted hepatocellular carcinoma cells upon Sorafenib treatment. Taken together, our findings demonstrated that USP22 inhibits Sorafenib‐induced ferroptosis through the transcriptional inhibition of *TFRC*.

### 2.8 USP22 depletion inhibits hepatocellular carcinoma growth and sensitises hepatocellular carcinoma to Sorafenib treatment in mice

**FIGURE 8:**
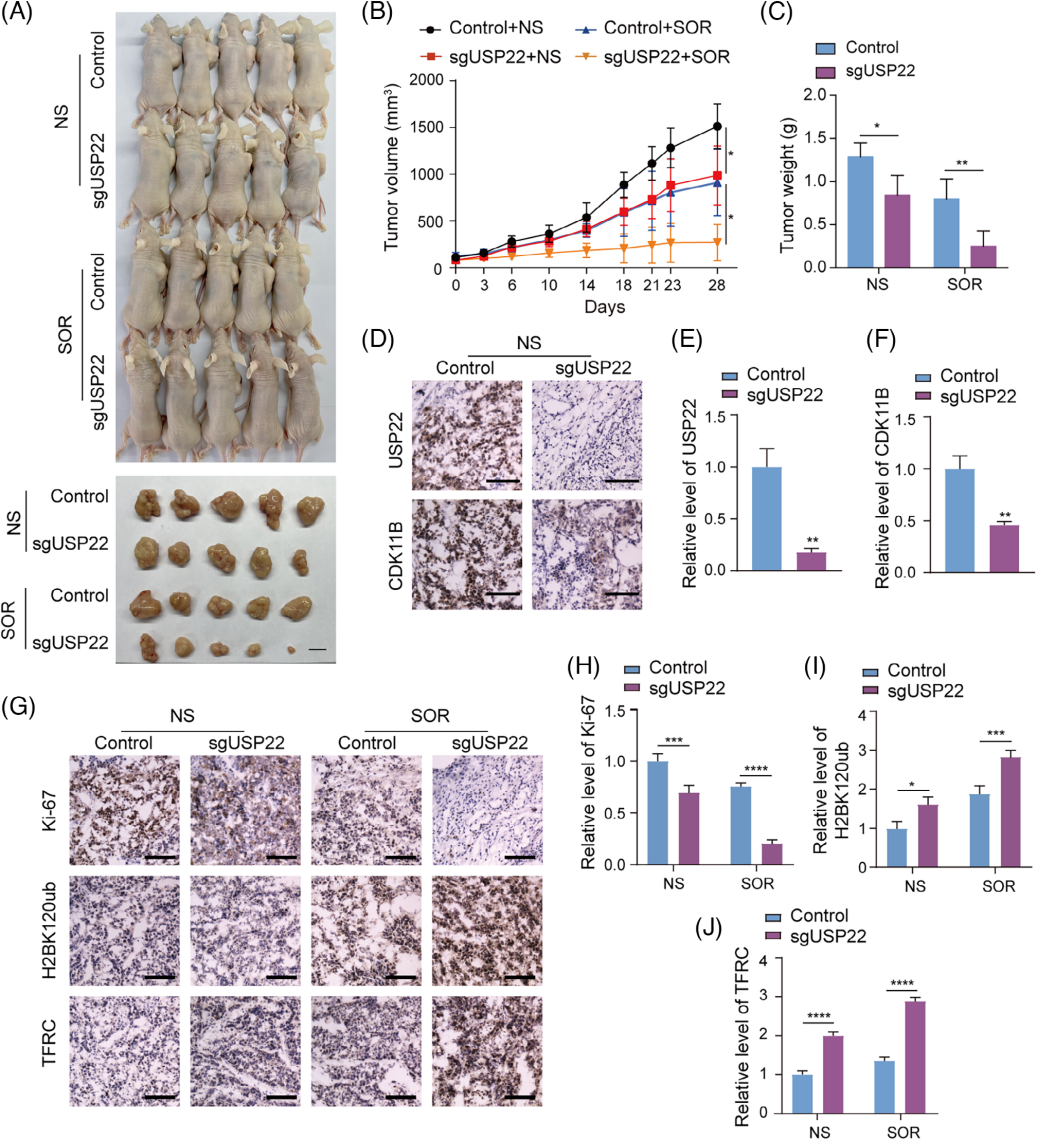
Ubiquitin‐specific protease 22 (USP22) depletion inhibits hepatocellular carcinoma growth and sensitises hepatocellular carcinoma to Sorafenib treatment in mice. (A) Control HepG2 cells or *USP22* knockout HepG2 cells were transplanted into female athymic nude mice. When the tumour volume reached approximately 80 mm^3^, mice were administered by gavage with vehicle (physiological saline) or Sorafenib (20 mg/kg/day). Tumours were stripped out 27 days later and photographed. (B) Tumors were measured at indicated time since drug treatment using a Vernier caliper and the volume was calculated according to the formula: *V* = π/6 × length × width^2^. Data are mean ± SD for *n* = 5; ^*^ *p* < .05 (two‐way ANOVA followed by Tukey's test for multiple comparisons). (C) Tumours were weighed. Data are mean ± SD for *n* = 5; ^*^ *p* < .05, ^**^ *p *< .01 (two‐way ANOVA followed by Tukey's test for multiple comparisons). (D) Immunohistochemistry was used to detect the protein levels of USP22 and cyclin‐dependent kinase 11B (CDK11B) in tumour tissues from mice treated with normal saline. Scale bar: 50 µm. (E and F) Quantitative analysis of immunohistochemistry staining in figure (D). Data are mean ± SD for *n* = 3; ^**^ *p *< .01 (Student's *t*‐test). (G) Immunohistochemistry was used to detect the levels of Ki‐67, H2BK120ub and transferrin receptor (TFRC) in subcutaneous tumour tissues. Scale bar: 50 µm. (H‒J) Quantitative analysis of immunohistochemistry staining in figure (G). Data are mean ± SD for *n* = 3; ^*^ *p *< .05, ^***^ *p *< .001, ^****^ *p *< .0001 (two‐way ANOVA followed by Tukey's test for multiple comparisons).

We have demonstrated that USP22 depletion inhibits hepatocellular carcinoma cell proliferation and promotes the sensitivity of hepatocellular carcinoma cells to Sorafenib‐induced ferroptosis by in vitro assays. A subcutaneous tumour model in nude mice was then applied to investigate the in vivo effect of USP22 depletion on hepatocellular carcinoma growth and response to Sorafenib. Control or USP22‐depleted HepG2 cells were subcutaneously injected into nude mice. Once the tumour volume reached approximately 80 mm^3^, mice in the control and knockout groups were treated with either normal saline or Sorafenib (20 mg/kg/day) by oral gavage on a daily basis. The growth of the implanted tumours was measured. After 27 days, the mice were euthanised, and the tumours were excised, weighed and photographed (Figure 8A). Our results showed a significant suppression of tumour growth in mice receiving *USP22* knockout tumours, particularly under Sorafenib treatment, as evident from the reduced tumour volume and weight in the USP22‐depleted groups (Figure 8B,C). The knockdown of USP22 expression in the xenograft was confirmed by immunohistochemistry (IHC) staining of USP22 in frozen sections of the tumours (Figure 8D,E). Consistent with the results at the cellular level, the protein level of CDK11B was reduced in *USP22* knockout tumour tissues compared to the control group (Figure 8D,F). In addition, IHC staining of Ki‐67, H2BK120ub and TFRC in frozen sections from all four groups indicated that *USP22* knockout promoted H2BK120ub and TFRC levels in transplanted tumours, and inhibited hepatocellular carcinoma malignancy as indicated by Ki‐67 staining, especially after Sorafenib treatment (Figure 8G‒J). Altogether, these results provided evidence that USP22 promotes the proliferation of hepatocellular carcinoma cells by stabilising CKD11B and increases Sorafenib resistance through reducing H2BK120ub level and *TFRC* transcription in vivo.

## 3 DISCUSSION

The low survival rate of hepatocellular carcinoma is due to the lack of diagnostic biomarkers and molecular therapeutic targets. Sorafenib is a first‐line systemic treatment for advanced hepatocellular carcinoma; however, drug resistance is a main issue. In this study, we found that USP22 is critically involved in the proliferation and drug resistance of hepatocellular carcinoma cells. USP22 promotes the proliferation of hepatocellular carcinoma cells by stabilising CDK11B and enhances resistance to Sorafenib by inhibiting ferroptosis through the USP22/H2BK120ub/TFRC axis.

So far, more than 40 USPs have been directly or indirectly associated with relevant cancer processes.^44^ Among them, USP7 has been widely implicated in various human cancers, primarily due to its well‐characterised role in regulating the MDM2/MDMX‐p53 circuitry.^45^, ^46^ USP16 affects cancer cell proliferation by deubiquitinating H2AK119, thereby influencing chromatin remodelling,^47^, ^48^, ^49^ and by deubiquitinating non‐histone substrates such as c‐Myc^50^ and Polo‐like kinase 1 (PLK1),^48^ which are critical for cell cycle progression. Notably, USP22 has been reported to participate in cell cycle regulation, invasion and metastasis, immune regulation, stemness maintenance and chemoresistance of types of tumour cells.^51^ Our study has further reinforced the role of USP22 as a contributor to hepatocellular carcinoma progression and Sorafenib resistance. And these two functions are, respectively, achieved by deubiquitinating non‐histone substrate CDK11B and histone substrate H2BK120ub.

As a current standard first‐line treatment drug for advanced hepatocellular carcinoma, Sorafenib resistance remains a significant challenge in clinical cancer treatment. Notably, Sorafenib is a potent inducer of ferroptosis. Therefore, promoting Sorafenib‐induced ferroptosis might eliminate resistance and improve Sorafenib efficacy.^12^ Recent findings suggest that leukaemia inhibitory factor receptor sensitises hepatocellular carcinoma to Sorafenib treatment through regulating the expression of iron‐sequestering cytokine lipocalin 2 (LCN2).^52^ Yes1‐associated transcriptional regulator (YAP1) and WW domain‐containing transcription regulator 1 (WWTR1, also called TAZ), well‐characterised transcriptional effectors of Hippo signalling, have also been implicated as key factors in Sorafenib resistance in hepatocellular carcinoma by increasing SLC7A11 expression.^53^ Meanwhile, coactivator‐associated arginine methyltransferase 1 (CARM1) has also been identified as a critical suppressor of ferroptosis in our previous study, blocking Sorafenib‐induced ferroptosis by transcriptionally activating GPX4.^54^ In this study, we demonstrated USP22 as a novel driver of Sorafenib resistance in hepatocellular carcinoma, presenting a new potential therapeutic target for Sorafenib‐resistant hepatocellular carcinoma.

Histone ubiquitination primarily occurs in the monoubiquitinated form, with 5%–15% of H2A and 1% of H2B, leading to H2AK119ub and H2BK120ub, respectively.^55^ USP22 has been shown to remove the mono‐ubiquitin moieties from H2BK120ub and H2AK119ub.^14^ H2BK120ub is believed to enhance transcription elongation and interact with RNA polymerase II as part of the core elongation complex,^41^ while H2AK119ub is associated with transcriptional repression.^56^ Therefore, USP22 regulates gene expression by modulating the levels of H2AK119ub and H2BK120ub. In this study, we found that USP22 depletion in the presence of Sorafenib increased the level of H2BK120ub but not H2AK119ub, indicating H2BK120ub as a regulator of USP22‐mediated Sorafenib resistance. USP22 inhibits Sorafenib‐induced ferroptosis through transcriptional suppression of *TFRC* mediated by deubiquitinating H2BK120ub. Why USP22 has a preference for H2BK120ub as its substrate under Sorafenib treatment is worth further research.

In summary, our study demonstrated that USP22 promotes the growth of hepatocellular carcinoma and resistance to Sorafenib‐induced ferroptosis by removing ubiquitin moieties from non‐histone protein CDK11B and histone H2B. Our findings suggest USP22 as a promising prognostic biomarker and therapeutic target for hepatocellular carcinoma patients, particularly those with Sorafenib resistance. The small molecule inhibitors targeting USP22 can be explored in future to function alone or synergistically with Sorafenib to treat hepatocellular carcinoma.

## 4 MATERIALS AND METHODS

### 4.1 Cells and reagents

HepG2, Hep3B, Huh7 and HEK‐293FT cells were maintained in Dulbecco's modified eagle medium (Meilun Biotechnology Co., Ltd.) supplemented with 10% foetal bovine serum (Biological Industries) at 37°C with 5% CO_2_. All cells were regularly authenticated by morphological observation and tested for mycoplasma contamination.

Antibodies and reagents were purchased from the following sources: anti‐USP22 (ab195289), anti‐histone H2A (ab177308) and anti‐histone H2B (ab52484) from Abcam Ltd.; anti‐CDK11B (A12830), anti‐TFRC (A5865) and anti‐β‐actin (AC038) from ABclonal Technology Co., Ltd.; anti‐H2BK120ub (5546), anti‐His (12698), anti‐Ki67 (9027) and anti‐GAPDH (2118) from Cell Signalling Technology Inc.; anti‐H2AK119ub (PTM‐1121) from PTM Biolabs Inc.; anti‐ubiquitin (U7258) from Sigma‒Aldrich Corp.; anti‐Flag (M2, F3165) from Merck KGaA Co.; horseradish peroxidase (HRP)‐conjugated secondary antibodies (5220‐0341 and 5220‐0336) from SeraCare Life Sciences Inc.; fluorescein‐conjugated (111‐095‐003) or rhodamine‐conjugated (111‐025‐003 and 115‐025‐003) secondary antibodies from Jackson ImmunoResearch Laboratories Inc.; Sorafenib (HY‐10201, final concentration 10 µM), Erastin (HY‐15763, final concentration 10 µM), RSL3 (HY‐100218A, final concentration 1 µM) and DFO (HY‐D0903, final concentration 3 µM) from MedChemExpress; Fer‐1 (T6500, final concentration 5 µM) from Target Molecule Corp.; CHX (S7418, final concentration 50 µM) and MG132 (S2619, final concentration 10 µM) from Selleck Chemicals.

### 4.2 Gene knockdown/knockout/overexpression cell line generation and plasmids transfection

HepG2, Hep3B and Huh7 cells with stable knockout of *USP22*, stable knockdown of *USP22* or *CDK11B*, or stable overexpression of *USP22* were established by lentiviral infection. Briefly, a sgRNA targeting *USP22* (CTTTGTCATAGATGTAGTCC) was cloned into the Cas9‐expressing lentiviral vector CRISPR v2. siRNA sequence targeting *UPS22* (5′‐AGCTACCAGGAGTCCACAAAG‐3′) or *CDK11B* (5′‐CGGAAACGACATCGAGAAGAA‐3′) was cloned into the pLKO.1 lentiviral vector. A Flag tag coding sequence fused with the full‐length cDNA of *USP22* was cloned into the pCDH‐CMV‐MCS‐EF1‐Puro lentiviral vector. These lentiviral constructs were, respectively, co‐transfected with packaging vectors (psPAX2 and pMD2.G) into HEK‐293FT cells using polyethylenimine (PEI, 23 966, Polysciences). The supernatant of HEK‐293FT was collected after 24 and 48 h, filtered through a 0.45 µm pore size filter, and applied to infect HepG2, Hep3B and Huh7 cells. Stable cell lines were selected with 1 µg/mL puromycin.

The cDNA sequences of USP22 fragments 1–160 aa and 161–525 aa were amplified by PCR and cloned into the pCDH‐CMV‐MCS‐EF1‐Puro vector. For transient transfection, HEK‐293FT cells were transfected with plasmids using PEI. siRNAs against *TFRC* (5′‐UGGUCAGUUCGUGAUUAAATT‐3′) and control siRNAs were purchased from GenePharma Co., Ltd. and transfected into HepG2 and Hep3B cells using Lipofectamine RNAiMAX (13778150, Thermo Fisher).

### 4.3 MTT assay

Cell viability was assessed using the MTT assay. Cells were seeded in 96‐well plates at approximately 1000 cells per well. Twenty‐four hours later, MTT solution (M8180, Solarbio, final concentration is 0.5 mg/mL) was replaced into each well for a 4‐h incubation. After removal of the MTT solution, 110 µL DMSO was added to dissolve the formazan product, and the absorbance was spectrophotometrically measured at 490 nm using a microplate reader.

### 4.4 Colony formation assay

Cells were seeded in six‐well plates at 500 cells per well. The culture medium was refreshed every 3 days. After 2 weeks, the medium was removed, and the cells were fixed with 4% paraformaldehyde for 15 min, followed by staining with 0.1% crystal violet solution (Solarbio) for 10 min. Images of the colonies were captured, and the colony numbers were counted using ImageJ software.

### 4.5 EdU‐incorporation assay

The EdU‐incorporation assay was performed using BeyoClick EdU Cell Proliferation Kit with Alexa Fluor 594 or Alexa Fluor 488 (C0078S or C0071S, Beyotime) according to the manufacturer's instructions. Briefly, cells were seeded in 96‐well plates at a density of 3000 cells per well. After 24 h of incubation, cells were cultured with EdU solution (EdU final concentration is 10 µM) for 2 h and then fixed with 4% paraformaldehyde for 15 min. Following rinsing with washing buffer (3% bovine serum albumin [BSA] in PBS), cells were permeabilised with 0.3% Triton X‐100 in PBS for 10 min and treated with 100 µL of click reaction solution for 30 min. Subsequently, Hoechst 33342 was added to stain the cell nuclei for 10 min in the dark, and images were captured using a fluorescence microscope.

### 4.6 Flow cytometry analyses of cell cycle

Cells were harvested, washed twice with PBS, and fixed in 70% ethanol at 4°C overnight. Then, the fixed cells were washed with PBS and incubated with 1 mg/mL RNase for 30 min to remove RNA. Subsequently, the cells were stained with PI at room temperature in the dark for 30 min to label DNA. Finally, the cell cycle distribution was analysed using a flow cytometer (BD FACSVerse), and the data were analysed using FlowJo software.

### 4.7 Immunofluorescent staining

Cells expressing Flag‐USP22 or His‐GFP‐CDK11B were plated on glass coverslips. After attachment, cells were fixed with 4% paraformaldehyde for 8 min, permeabilised with 0.2% Triton X‐100 for 5 min, and blocked with 2% BSA for 30 min. The coverslips were then incubated with the primary antibodies overnight at 4°C, followed by incubation with fluorescein‐ or rhodamine‐conjugated secondary antibodies for 1 h. Nuclei were stained with 0.5 µg/mL 4′,6‐diamidino‐2‐phenylindole for 5 min in the dark. Coverslips were mounted with Fluorescence Mounting Medium (S302380, Agilent), and images were captured by a confocal microscope.

### 4.8 Flag affinity purification

HEK‐293FT cells were transfected with either control vectors or Flag‐USP22 expression constructs for 48 h and lysed in cell lysis buffer (50 mM Tris‒HCl, pH 7.4, 150 mM NaCl, 1 mM Ethylenediaminetetraacetic acid [EDTA] and 0.5% Triton X‐100) supplemented with protease inhibitors (B14001, Selleck) on ice for 30 min. The supernatant was obtained by centrifugation at 12 000 rpm at 4°C for 20 min. Anti‐Flag M2 gel (A2220, Sigma‒Aldrich) was incubated with the supernatant at 4°C overnight. After thorough washes, the bound proteins were eluted using Flag peptide, and subjected to SDS‒PAGE. Unique protein bands identified by silver staining in the Flag‐USP22 overexpression group were excised from the gel and analysed by liquid chromatography‒mass spectrometry.

### 4.9 Co‐immunoprecipitation

Cells were lysed in cell lysis buffer (50 mM Tris‒HCl, pH 7.4, 150 mM NaCl, 1 mM EDTA and 0.5% Triton X‐100) supplemented with protease inhibitors at 4°C for 30 min. After centrifugation at 12 000 rpm for 10 min, the supernatant was incubated with the primary antibodies or normal immunoglobulin G overnight at 4°C, followed by incubation with Protein A/G beads (B23201, Bimake) for another 2 h. Immunoprecipitated beads were washed three times with washing buffer (50 mM Tris‒HCl, pH 7.4, 150 mM NaCl, 1 mM EDTA and 0.1% Triton X‐100), and boiled with 2 × loading buffer at 100°C for 10 min. The supernatants were then subjected to Western blotting.

### 4.10 Calcein‐AM/PI staining

Live/dead cell staining was performed using a Calcein‐AM/PI Double Staining Kit (C542, Dojindo). Cells were seeded in 6‐cm dishes at a density of 1 × 10^5^ cells per dish overnight and treated with DMSO or Sorafenib (10 µM) for 24 h. Then, the cells were collected by trypsin digestion, washed twice with PBS buffer, and stained using a mixture of Calcein‐AM and PI solution for 15 min at 37°C. Fluorescence images were captured using a fluorescence microscope, and the percentage of dead cells was calculated.

### 4.11 Reduced GSH measurement

Cells were seeded in 10‐cm dishes at a density of 2 × 10^6^ cells per dish and cultured for 24 h. Following treatment with either DMSO or Sorafenib for an additional 24 h, cells were harvested and counted. Intracellular levels of reduced GSH were quantified using the Reduced GSH Assay Kit (BC1175, Solarbio) in accordance with the manufacturer's protocol. The concentration of reduced GSH was determined using a standard curve and normalised to the cell number.

### 4.12 Lipid peroxidation measurement

Lipid peroxidation was assessed using the fluorescent probe BODIPY 581/591 C11 (D3861, Thermo Fisher). Approximately 2 × 10^6^ HepG2 cells were plated in a 10‐cm dish and cultured overnight. After the designated treatments, cells were collected and incubated with BODIPY 581/591 C11 at a final concentration of 5 µM for 30 min in the dark at 37°C. Cells were then washed twice with PBS, and lipid peroxidation levels in the cell suspensions were measured by a flow cytometer.

### 4.13 Intracellular Fe^2+^ measurement

To determine the concentrations of ferrous iron (Fe^2+^) within cells, FerroOrange (F374, Dojindo) was utilised. Cells were seeded at a density of 4000 cells per well in 96‐well plates and cultured overnight. After 24 h of treatment with either Sorafenib or DMSO, cells were washed twice with serum‐free medium and incubated with serum‐free medium containing 1 µM FerroOrange at 37°C for 30 min. Fluorescence images were captured using a fluorescence microscope. Fe^2+^ levels were quantified using ImageJ software.

### 4.14 Chromatin‐immunoprecipitation

Cells were cross‐linked with 1% formaldehyde for 10 min and quenched with 0.125 M glycine. After washing twice with PBS, cells were lysed in SDS buffer (50 mM Tris‒HCl, pH 8.0, 100 mM NaCl, 5 mM EDTA and 10% SDS) supplemented with protease inhibitors, and then centrifugated at 1200 rpm for 10 min. Cell pellets were resuspended in IP buffer (100 mM NaCl, 66.67 mM Tris‒HCl, pH 8.0, 5 mM EDTA, 0.33% SDS and 1.67% Triton X‐100), and chromatin was sonicated to generate DNA fragments. For Spike‐in ChIP‐seq, *Drosophila Schneider* 2 cells (S2 cells) were prepared and processed in the same manner. Depending on the DNA concentration of each sample, chromatin from HepG2 cells was mixed with S2 cell chromatin at a ratio of 5:1. Subsequently, the chromatin was incubated with the indicated antibodies overnight at 4°C, and precipitated using Protein A/G beads for 4 h. Beads were washed three times with wash buffer 1 (20 mM Tris‒HCl, pH 8.0, 150 mM NaCl, 2 mM EDTA, 0.1% SDS and 1% Triton X‐100), and once with wash buffer 2 (20 mM Tris‒HCl, pH 8.0, 500 mM NaCl, 2 mM EDTA, 1% Triton X‐100 and 0.1% SDS). Reverse crosslinking was performed at 65°C for 6 h. For ChIP‐qPCR, purified DNA was used for qPCR and primers are listed in Table S1. For Spike‐in ChIP‐seq, purified DNA was subjected to high throughput sequencing by Novogene Bioinformatics Technology Co., Ltd.

For Spike‐in ChIP‐seq analysis, reads were aligned separately to the human reference genome (hg38) and the Drosophila melanogaster reference genome (dm3) using Bowtie2 (v2.4.5). PCR duplicates were removed by Sambamba (v1.0). To normalise the data, a scaling factor was determined based on the number of Spike‐in S2 reads, and Spike‐in normalised bigwig files were generated using the deepTools (v3.5.4) bamCoverage function. Peaks of H2BK120ub were called using MACS2 (v2.2.9.1) callpeak function, and differential peaks were detected using the MACS2 bdgdiff function. For downstream analyses, heatmaps and plot profiles were generated with deepTools computeMatrix and plotHeatmap/plotProfile functions, respectively, using Spike‐in normalised bigwig files. Differential peaks annotation was performed using the ChIPSeeker (R package v1.30.3) annotatePeak and plotAnnoPie functions.

### 4.15 RNA‐seq

Stable *USP22* knockout HepG2 cells or control cells were treated with 10 µM Sorafenib for 24 h. Total RNA was extracted by TRIzol (P118, GenStar), and submitted for RNA‐seq by BGI Genomics Co., Ltd. Reads were aligned to the human reference genome (hg38) using HISAT2 (v2.2.1). Samtools (v1.6) was used to sort and set the index of mapped results and then transfer them from sam to bam format. Gene counts and transcript counts were calculated using StringTie (v2.2.1). Differentially expressed genes (DEGs) were identified using DESeq2 (R package v1.34.0). DEGs were identified according to padj < .05 and fold change ≥ 1.5.

### 4.16 RNA isolation and quantitative real‐time RT‐PCR

Total RNA was isolated from cells using TRIzol reagent (P118, GenStar), and reverse transcribed into cDNA using Hifair III 1st Strand cDNA Synthesis SuperMix for qPCR (11141ES60, YEASEN). qPCR was conducted with RealStar Power SYBR qPCR Mix (A311, GenStar). Relative mRNA expression was calculated using the 2^−ΔΔCt^ method and normalised to GAPDH, an internal control. Primers for RT‐qPCR are listed in Table S1.

### 4.17 Animal experiments

Animal studies were approved by the Institutional Animal Care and Use Committees of Tianjin Medical University. For cell line‐derived xenografts models, 5 × 10^6^ stable *USP22* knockout HepG2 cells or control cells were suspended in 100 µL PBS, mixed with Matrigel (1:1 volume), and subcutaneously injected into the right flank of the female athymic nude mice (BALB/c; 4–5 weeks of age; 5 mice per group) (Charles River Laboratories). Once the tumour volume reached approximately 80 mm^3^, mice were randomly divided into designated groups and treated with vehicle (physiological saline) or Sorafenib (20 mg/kg/day) via oral gavage. The tumour sizes were measured with a Vernier caliper and calculated using the formula: *V* = π/6 × length × width^2^. After 27 days, the mice were euthanised, and the tumours were resected and weighed.

### 4.18 Immunohistochemistry

The resected tumours from mice were embedded in optimal cutting temperature compound (#4583, Sakura), frozen at ‒80°C and sectioned into 8 µm sections, which were subsequently stored at ‒80°C. For IHC, endogenous peroxidase activity was quenched with 3% hydrogen peroxide, and non‐specific signals were blocked with PBST (PBS with 0.1% Triton X‐100) containing 10% goat serum. The slides were then incubated with primary antibodies overnight at 4°C, followed by incubation with secondary antibodies conjugated to HRP at room temperature for 1 h. Colour development was achieved using a diaminobenzidine substrate kit (ZLI‐9017, ZSGB‐BIO), and the slides were counterstained with haematoxylin (G1120, Solarbio). Images were captured using a microscope. IHC staining intensity was scored on a scale from 0 (negative) to 10 (strongest positive) and evaluated by two independent investigators.

### 4.19 Statistical analysis

All data are expressed as mean ± SD from at least three independent experiments. Statistical analyses were performed using GraphPad Prism 9.0. Student's *t*‐test was used to evaluate the statistical significance between two groups, one‐way ANOVA followed by Tukey's multiple comparison was performed to assess differences among multiple groups, and two‐way ANOVA followed by Tukey's multiple comparison was used for comparisons involving groups with and without Sorafenib treatment. A *p*‐value of < .05 was considered statistically significant.

## AUTHOR CONTRIBUTIONS

Chenghao Xuan conceived and supervised the project, and wrote and revised the manuscript. Xiaochen Wang and Yijie Su performed most of the experiments. Xuanyuan Li conducted the bioinformatics analysis. Bodi Zhang performed some experiments. Liang Zhang performed the transmission electron microscopy. Xiaochen Wang and Bei Lan checked the original data and wrote the manuscript. Yingmei Wang and Chunze Zhang revised the manuscript.

## CONFLICT OF INTEREST STATEMENT

The authors declare they have no conflicts of interest.

## ETHICS STATEMENT

The institutional Animal Care and Use Committees of Tianjin Medical University gave the approval for all animal studies.

## Supporting information



Supporting Information

## Data Availability

The raw and processed high‐throughput sequencing data (RNA‐seq and ChIP‐seq) were deposited in the Gene Expression Omnibus database under accession numbers GSE278684 and GSE278685, respectively.
