# Supplementary material for: USP22 promotes the proliferation and Sorafenib resistance of hepatocellular carcinoma cells via its deubiquitinase activity
Source: Clin Transl Med. 2025 May 7;15(5):e70324. doi: 10.1002/ctm2.70324 (PMC12059209; doi:10.1002/ctm2.70324)
Supplement: Supplementary file 1 — Supporting Information [file CTM2-15-e70324-s001.pdf]

Supplementary Table 1. Primer sequences used in this study.

RT-qPCR primer sequences (5'-3')		
	Forward	Reverse
CDK11B	CCGACTTACAGGACATCAGCGA	CTCCTCTGATTCTTCACTGGTGC
TFRC	ATCGGTTGGTGCCACTGAATGG	ACAACAGTGGGCTGGCAGAAAC
SLC9A9	CTGATGTCTGCTACAGATCCAGT	CCAGCGAAGATTCCCAGGAAA
SCARA5	AAAGCTATGTACCTACACACCGT	CCGCCGTTTGTGACATGGA
MDM2	CAATCAGCAGGAATCATCGG	GCTTCTTTCACAACATATCTCCC
BTG2	ACCACTGGTTTCCCGAAAAG	CTGGCTGAGTCCGATCTGG
FBXW7	ACTGGGCTTGTACCATGTTCA	TGAGGTCCCCAAAAGTTGTTG
GAPDH	ACCACAGTCCATGCCATCAC	TCCACCACCCTGTTGCTGTA

ChIP-qPCR primer sequences (5'-3')		
	Forward	Reverse
TFRC	ATCCTCTTATCAACGGGGAAG	ATTCTGATTCGGCTCTTTTCGG

Supplementary Figures

Supplementary Figure 1

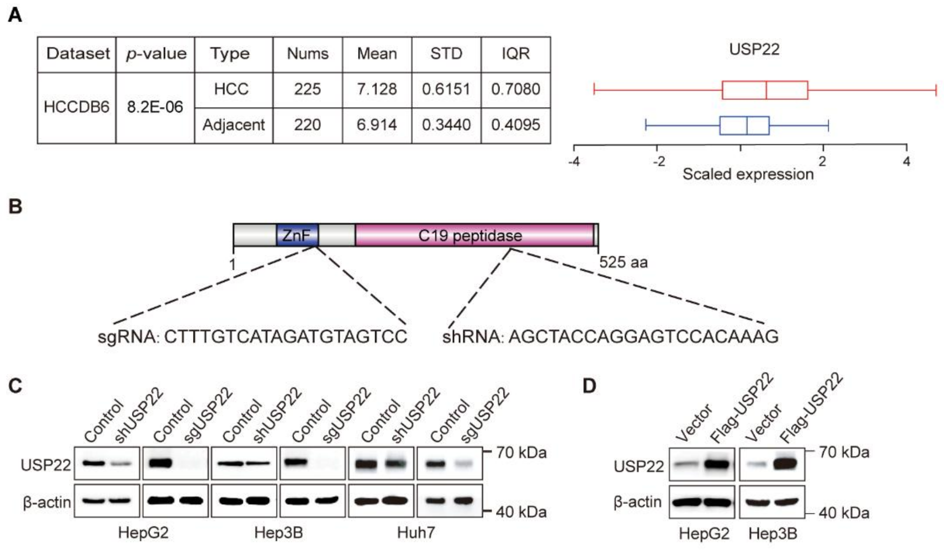

Supplementary Figure 1. Analysis of *USP22* mRNA levels in hepatocellular carcinoma samples from Chinese patients and generation of hepatocellular carcinoma cell lines stable with *USP22* knockdown/knockout/overexpression.

A. Analysis of *USP22* mRNA levels in 225 hepatocellular carcinoma samples and 220 adjacent non-tumor samples from Chinese hepatocellular carcinoma patients using HCCDB6 dataset from the HCCDB v2.0 database (http://lifeome.net/database/hccdb2). $p < 0.0001$ (*t*-test). **B.** Schematic diagram of USP22 sgRNA/shRNA target sites. **C.** Western blotting was used to detect the expression of USP22 in HepG2, Hep3B, and Huh7 cells stably expressing *USP22* shRNA/sgRNA or not. β-actin was used as an internal control. **D.** Cell lysates of HepG2 and Hep3B cells stably overexpressing *USP22* or not were subjected to Western blotting.

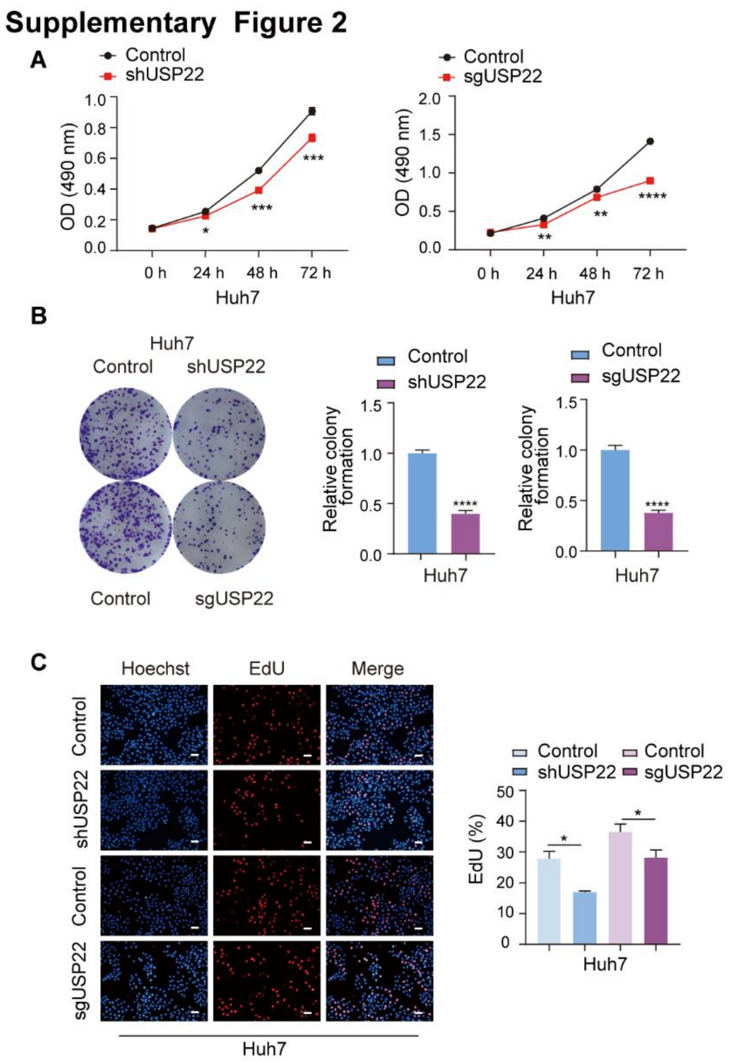

Supplementary Figure 2. USP22 knockdown/knockout inhibits the proliferation of Huh7 cells.

A-C MTT assay (A), colony formation assay (B), and EdU-incorporation assay (C) were performed in the indicated cells. Data are mean ± SD for n = 3; *$p < 0.05$, **$p < 0.01$, ***$p < 0.001$, ****$p < 0.0001$ (Student's *t*-test).

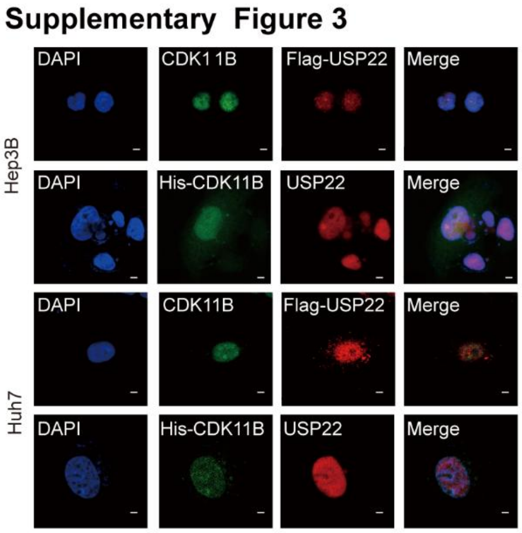

Supplementary Figure 3. USP22 and CDK11B are co-localized in the nucleus.

Hep3B/Huh7 cells were transfected with Flag-USP22 or His-GFP-CDK11B plasmid, and the subcellular localizations of CDK11B and USP22 were detected by immunofluorescence staining with anti-Flag plus anti-CDK11B, or with anti-USP22. Nuclei were visualized by DAPI staining. Scale bar: 5 μm.

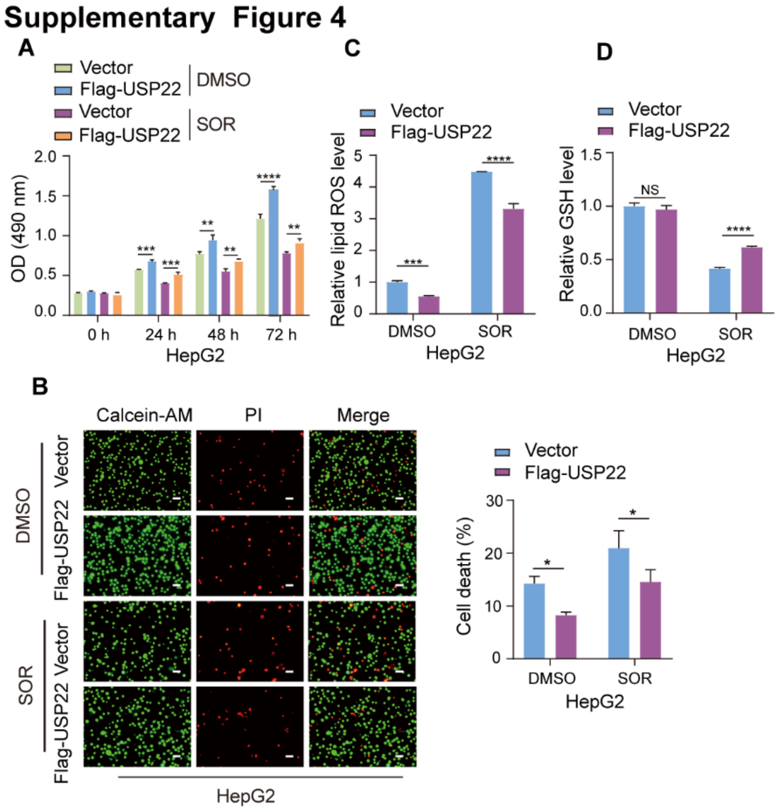

Supplementary Figure 4. USP22 overexpression inhibits sorafenib-induced ferroptosis in HepG2 cells.

A. HepG2 cells with *USP22* overexpression and control cells were treated with DMSO or sorafenib (10 μM) for the indicated times. Cell viability was monitored with MTT assays. **B.** Calcein-AM/PI staining was performed in the indicated cells. Representative images and quantitative analysis of cell death percentage are shown. Scale bar: 50 μm. **C-D.** The indicated cells were treated with DMSO or sorafenib (10 μM) for 24 hours. The levels of cellular lipid ROS (C) and GSH (D) were detected. For figures A-D, data are mean ± SD for n = 3; NS, $p > 0.05$, *$p < 0.05$, **$p < 0.01$, ***$p < 0.001$, ****$p < 0.0001$ (two-way ANOVA followed by Tukey's test for multiple comparisons).

Supplementary Figure 5

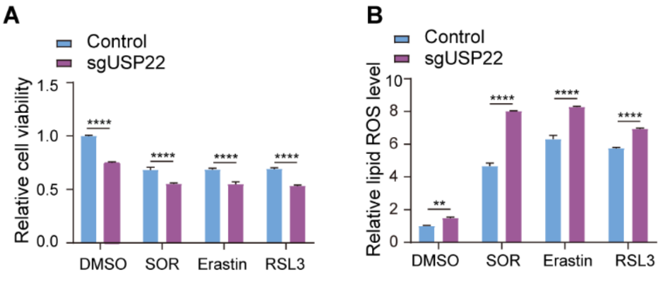

Supplementary Figure 5. USP22 depletion promotes ferroptosis induced by different ferroptosis inducers.

HepG2 cells with *USP22* knockout or not were treated with DMSO, sorafenib (10 μM), erastin (10 μM), or RSL3 (1 μM) for 24 hours. Cell viability (A) and cellular lipid ROS levels (B) were examined. Data are mean ± SD for n = 3; **$p < 0.01$, ****$p < 0.0001$ (two-way ANOVA followed by Tukey's test for multiple comparisons).
